# Kisspeptin Alleviates Human Hepatic Fibrogenesis by Inhibiting TGFβ Signaling in Hepatic Stellate Cells

**DOI:** 10.3390/cells13191651

**Published:** 2024-10-04

**Authors:** Kavita Prasad, Dipankar Bhattacharya, Shams Gamal Eldin Shams, Kimberly Izarraras, Tia Hart, Brent Mayfield, Maryjka B. Blaszczyk, Zhongren Zhou, Utpal B. Pajvani, Scott L. Friedman, Moshmi Bhattacharya

**Affiliations:** 1 Department of Medicine, Robert Wood Johnson Medical School, Rutgers University, New Brunswick, NJ 08901, USA; kp460@connect.rutgers.edu (K.P.); ss4459@rwjms.rutgers.edu (S.G.E.S.); ki113@gsbs.rutgers.edu (K.I.); tmh187@cinj.rutgers.edu (T.H.); 2 Division of Liver Diseases, Icahn School of Medicine at Mount Sinai, New York, NY 10029, USA; dipankar.bhattacharya@mssm.edu (D.B.); scott.friedman@mssm.edu (S.L.F.); 3 Department of Medicine, Columbia University, New York, NY 10032, USA; bcm2151@cumc.columbia.edu (B.M.); up2104@cumc.columbia.edu (U.B.P.); 4 Department of Pathology and Laboratory Medicine, Robert Wood Johnson Medical School, Rutgers University, New Brunswick, NJ 08901, USA; mb2001@rwjms.rutgers.edu (M.B.B.); zz442@rwjms.rutgers.edu (Z.Z.)

**Keywords:** KISS1R, kisspeptin, MASLD, MASH, fibrosis, hepatic stellate cells, TGFβ

## Abstract

The peptide hormone kisspeptin attenuates liver steatosis, metabolic dysfunction-associated steatohepatitis (MASH), and fibrosis in mouse models by signaling via the kisspeptin 1 receptor (KISS1R). However, whether kisspeptin impacts fibrogenesis in the human liver is not known. We investigated the impact of a potent kisspeptin analog (KPA) on fibrogenesis using human precision-cut liver slices (hPCLS) from fibrotic livers from male patients, in human hepatic stellate cells (HSCs), LX-2, and in primary mouse HSCs. In hPCLS, 48 h and 72 h of KPA (3 nM, 100 nM) treatment decreased collagen secretion and lowered the expression of fibrogenic and inflammatory markers. Immunohistochemical studies revealed that KISS1R is expressed and localized to HSCs in MASH/fibrotic livers. In HSCs, KPA treatment reduced transforming growth factor b (TGFβ)-the induced expression of fibrogenic and inflammatory markers, in addition to decreasing TGFβ-induced collagen secretion, cell migration, proliferation, and colony formation. Mechanistically, KISS1R signaling downregulated TGFβ signaling by decreasing SMAD2/3 phosphorylation via the activation of protein phosphatases, PP2A, which dephosphorylates SMAD 2/3. This study revealed for the first time that kisspeptin reverses human hepatic fibrogenesis, thus identifying it as a new therapeutic target to treat hepatic fibrosis.

## 1. Introduction

Hepatic fibrosis is characterized by the excessive deposition of extracellular matrix proteins, such as type 1 collagen, leading to distorted hepatic architecture. Fibrosis results from the activation of hepatic stellate cells (HSCs), which are the major fibrogenic cell type in the liver. Activated HSCs acquire the characteristics of mesenchymal fibroblast cells and produce various extracellular matrix constituents, such as collagen, fibronectin, and α-smooth muscle actin (α-SMA) [1,2]. The liver can develop cirrhosis and eventually ceases to function due to excessive scar production. Some patients can progress to hepatocellular carcinoma (HCC) [3,4], a fatal cancer that is on the rise [5]. Hepatic fibrosis results from chronic injuries to the liver, such as those caused by alcohol, hepatitis, and metabolic dysfunction-associated steatohepatitis (MASH), previously known as non-alcoholic steatohepatitis (NASH). MASH is characterized by hepatocyte inflammation and injury due to deregulated fat metabolism and is an advanced form of metabolic dysfunction-associated steatotic liver disease (MASLD). Fibrosis predicts mortality and disease severity [6] in females; MASH is now the leading cause of liver transplant and second to alcoholic liver disease in males [7]. Thus, there is a direct need for the development of new therapeutics to treat fibrosis. 

Kisspeptins are peptide hormones, encoded by the *KISS1* gene, that circulate in the blood and bind to and signal via the G_αq/11_ protein-coupled receptor, kisspeptin 1 receptor (KISS1R) [8,9]. The kisspeptin/KISS1R signaling system is expressed centrally in the brain and plays a key role in regulating puberty and reproduction in addition to metabolism [8,10,11]. Peripherally, KISS1 and KISS1R are expressed in several metabolic tissues, including the pancreas, adipose tissue, and liver [8,12]. The liver produces kisspeptin, which has been shown to be a major regulator of insulin resistance in type-2 diabetes and gestational diabetes [13,14,15,16,17]. We have shown using a pre-clinical mouse model of MASH/fibrosis that kisspeptin administration reduces advanced (F3) hepatic fibrosis [13] in mice in a Diet-Induced Animal Model of Non-Alcoholic Liver Disease (DIAMOND) [18] and decreased serum alanine aminotransferase (ALT) levels, which is a clinical biomarker for MASH that is indicative of liver injury [13]. Importantly, kisspeptin treatment of DIAMOND mice fed a high-fat ‘Western’ diet (HFD) and sugar water exhibited a significant reduction in several inflammatory and profibrogenic markers [13], including transforming growth factor β1 (TGFβ1), a critical mediator of hepatic fibrosis [19,20] and decreased hepatic hydroxyproline levels (indicative of collagen content) [13]. However, whether the activation of KISS1R directly regulates fibrogenesis is not known. In this work, we tested the hypothesis that kisspeptin directly attenuates pathways regulating HSC activation to mediate its anti-fibrotic effects. We observed that KISS1R is expressed in HSCs in fibrotic livers from MASH patient biopsies. The effects of kisspeptin treatment were assessed in patients with fibrotic livers using the clinically relevant human precision-cut liver slices (hPCLS) model. In this model, the morphological (e.g., cell types, architecture, heterogeneity) and biological (e.g., extracellular matrix) organization of the native liver, as well as the pathological features of the disease, are preserved [21,22]. We demonstrate that kisspeptin treatment of patient hPLCS and human and mouse HSCs decreased fibrogenic and inflammatory markers via KISS1R-mediated downregulated TGFβ-induced signaling in HSCs.

## 2. Materials and Methods

### 2.1. Human Precision-Cut Liver Slices

Human precision-cut liver slices (hPCLS) were generated as previously described [23], from surgically resected de-identified human fibrotic livers from three male patients (Table 1) following Institutional Review Board (IRB) approval (HS#: 20-00485, dt. 7/31/2020) at Icahn School of Medicine at Mount Sinai, New York City, N.Y. In each case, fibrotic regions adjacent to tumors were selected from patients with HCC by a board-certified pathologist to generate 8 mm diameter and 200 µm thick liver slices. Fresh medium was added daily containing either a vehicle (PBS), TAK-448, a potent kisspeptin analog, henceforth referred to as KPA (3 nM, 100 nM), or 10 mM of TGFβ receptor 1 kinase inhibitor II (ALK5i), (Calbiochem, Burlington MA, USA, catalog #616452) [21] for the indicated times, after which the tissue was processed for measuring changes in protein expression using immunohistochemistry (see below). Additionally, total RNA was isolated from tissue using the RNeasy Mini Kit (Qiagen, Valencia CA, USA) and used for measuring changes in gene expression by RT-quantitative PCR (qPCR) as described [23] (see below). Secreted collagen was assessed in culture media by human pro-collagen I alpha 1 ELISA (Abcam, Cambridge, MA, USA ab210966) as described [23]. For cytotoxicity assessment, lactate dehydrogenase (LDH) and albumin levels were measured in the conditioned media, as previously described [23].

### 2.2. Human LX-2 Stellate Cell Culture and Treatment

LX-2 cells [24] were purchased from EMD Millipore (Burlington, MA, USA) and grown in Dulbecco’s modified Eagle’s medium (DMEM with 10 % (*v*/*v*) fetal bovine serum (FBS). Prior to treatment, cells were serum-starved overnight to synchronize metabolic activity in serum-free DMEM (Thermo Fisher Scientific, Waltham, MA, USA). Cells were treated every 24 h with KPA (3 nM, 100 nM), purchased from MedChem Express (Monmouth Junction, NJ, USA); these concentrations were selected based on previous studies [13,25]. KPA (3 nM) was added in the presence or absence of TGFβ (5 ng/mL) (R&D Systems, Minneapolis, MN, USA).

### 2.3. Primary Mouse Hepatic Stellate Cell (HSC) Culture and Treatment

Mice studies (protocol PROTO201702536, 19 July 2024) were approved by the Institutional Animal Care and Use Committee (IACUC) of Rutgers University. Primary HSCs were isolated as previously described [26,27] from C57Bl6/J mice. Cells (90,000/well) were grown in DMEM plus 10% FBS for 24 h, which was changed to serum-free medium containing KPA (3 nM, 48 h). RNA was isolated using the TRIzol reagent (Thermo Fisher, Waltham, MA, USA), and gene expression was determined as described [13] (see below) using the primers listed in Table S1.

### 2.4. Real-Time PCR (RT-qPCR) Quantification of Gene Expression

RT-qPCR was conducted using SYBR green (BioRad, Hercules CA, USA) RT-qPCR as described previously [13] (see Table S1 for primers). LX-2 cells (150,000 cells/well) were starved overnight and then treated for 48 h prior to RNA isolation using a RNeasy Mini Kit (Qiagen, Valencia, CA, USA).

### 2.5. RNA-Seq Data Analysis

LX-2 cells (150,000/well) were starved overnight and treated with either a vehicle (PBS), KPA (3 nM), and/or TGFβ (5 ng/mL) for 48 h. RNA was isolated (RNeasy Mini Kit Qiagen, Valencia, CA, USA) and assessed using the 2100 Bioanalyzer instrument (Agilent, Santa Clara, CA, USA) at the Albert Einstein College of Medicine Epigenomics Shared Facility. Libraries were made from purified RNA using the Qiaseq Stranded RNA lib Kit with UDI and QIAseq FastSelect -rRNA HMR Kit (Qiagen INC.) for Illumina sequencing. Libraries were QC using Fluorometric Quantitation (Qubit; Invitrogen: Thermo Fisher Scientific, Waltham, MA, USA), the Agilent 4150 TapeStation System, and QPCR (Roche Light Cycler). RNASeq libraries were multiplexed and sequenced as 1 × 100 bp single end on NEXTSEQ 2000 (Illumina NextSeq 500, Illumina.Inc, San Diego, CA, USA) following standard protocols. The sequencing files in FASTQ format for each sample were trimmed for adaptors using trim galore v0.3.7 and then aligned against human genome hg38 using STAR aligner v2.7.9a. Aligned sequencing data were then converted to gene count matrices using STAR. Differential gene analyses were performed using R package DESeq2 v1.42.0; genes with more than a 2-fold change and adjusted *p*-values below 0.05 were considered significantly different. Gene set enrichment analysis was conducted using R package fgsea v1.28.0 against KEGG and the GO database.

### 2.6. Immunoblot Analysis

For Western blot analysis, cells were lysed using RIPA with protease inhibitors as described [13,28,29]. Protein expression was determined using the following anti-human antibodies: anti-rabbit KISS1 (Protein Tech, Rosemont IL, USA catalog # 18375-1-AP; 1:1000), anti-rabbit GPR54 (Abcam, Cambridge, MA, USA catalog #ab137483; 1:1000), anti-mouse fibronectin (R&D Systems, Minneapolis, MN USA catalog # MAB19182; 1:1000). Antibodies from Cell Signaling Technologies (CST, Danvers, MA, USA): anti-rabbit collagen (CST catalog #91144-220; 1:1000), anti-rabbit snail (CST catalog #3895; 1:1000), anti-rabbit Phospho SMAD2/3 (CST catalog #8828) anti-rabbit SMAD2/3 (CST catalog # 8685) anti-mouse GAPDH (mAb catalog #97166), anti-rabbit SMA (CST catalog #19245), anti-mouse β-actin (CST catalog #3700T), and anti-rabbit vinculin (CST catalog #13901). Imaging was conducted using the Super Signal West Dura Extended Duration Substrate (catalog #34076 Thermo Scientific, Waltham, MA, USA) and ChemiDoc Touch imaging system (Bio-Rad, Hercules, CA, USA) and quantified using Image Lab Software (BioRad, Hercules, CA, USA). 

### 2.7. LX-2 Cell Proliferation Assay

Cell proliferation was determined using the BrdU Cell Proliferation Kit (catalog #126556 Abcam, Cambridge, MA, USA) [23]. Serum-starved cells (5000 cells/well) were plated in a 96-well plate and treated for 72 h, and then incubated with BrdU (2 h, 37 °C). Proliferation was quantified (absorbance measured at 370 nm with reference wavelength at 492 nm).

### 2.8. LX-2 Cell Transformation Assay

Cell transformation assays were performed using the soft-agar assay (3D) Cell Transformation Assay Kit (Colorimetric) (catalog #ab235698, Abcam, Cambridge, MA, USA). Cells (4 × 10^4^) were grown in soft agar for 7 days at 37 °C. Absorbance was measured at 450 nm upon the addition of the WST solution. 

### 2.9. Kisspeptin Secretion

Kisspeptin secretion assays were conducted as described [28]. Cells (5 × 10^5^ cells/well) were grown in phenol red-free RPMI with 10% FBS for 24 h. Conditioned media were used to assess secreted kisspeptin levels, using the Human KISS1 (68–121) Amide/Metastin (1–54) kit from Phoenix Pharmaceuticals Inc. Dayton, OH, USA, catalog #RK-04859. The protein concentration of cells per well was used to normalize secretion.

### 2.10. Immunohistochemistry of Human Liver

Immunohistochemistry was conducted on de-identified hPCLS, as described [23]. Briefly, sections (8 mm) were cut from fresh frozen tissue (OCT blocks) and fixed in methacarn (60% methanol, 30% chloroform and 10% acetic acid) for 15 min at −20 °C. After washing in TBS-T, sections were stained with a primary antibody using anti-collagen 1 (1:200, Rockland Immunochemicals Pottstown, PA, USA catalog #600-401-103-0.1) and anti α-SMA (1:200 Abcam, Cambridge, MA, USA Ab5694) overnight, followed by goat anti-rabbit Alexa Fluor 647 (1:500; Invitrogen#A21245) for 1 h. Sections were mounted using DAPI fluoromount-G (Southern Biotech, Birmingham, AB, USA catalog#0100-20).

Immunohistochemistry was conducted on formalin-fixed paraffin-embedded (FFPE) human liver sections as described [28,29]. The study was approved by Rutgers IRB (protocol Pro2019002570, 20 May 2020). De-identified FFPE sections from pathologist-verified MASH patients were obtained from archived liver tissue deposited at the Robert Wood Johnson University Hospital Anatomic Pathology laboratory. Following deparaffinization and heat-induced antigen retrieval, slides were incubated with primary antibodies: the rabbit anti-KISS1R (anti-GPR54: Abcam, Cambridge, MA, USA, ab137483; 1:250) and mouse anti-desmin (Abcam, Cambridge, MA, USA ab6322, 5 µg/mL) overnight. KISS1R immunoreactivity was detected using the Alexa Fluor 555 Tyramide SuperBoost Kit (ThermoFisher Scientific, catalog #B40923). Desmin immunoreactivity was detected using donkey anti-mouse Alexa Fluor 488 (1:500; Invitrogen catalog #A-21202). A matched negative staining control, a normal rabbit IgG polyclonal antibody (Sigma Aldrich, Burlington, MA, USA, catalog #12-370, 1:100), was used to confirm the primary antibody specificity. All slides were processed in parallel. Images were acquired using a Zeiss LSM 700 laser scanning microscope.

### 2.11. Statistical Analysis

The differences between experimental and treated groups were determined using unpaired, two-tailed Student’s t-test or one-way or two-way analysis of variance (ANOVA), followed by post hoc Bonferroni’s multiple comparison test (GraphPad Prism Software (version 10.3.1), Inc., La Jolla, CA, USA). All values are expressed as the mean ± SEM and a value of *p* < 0.05 was considered statistically significant.

## 3. Results

### 3.1. Kisspeptin Analog, TAK-448 (KPA), Reduces Fibrogenic and Inflammatory Markers as Well as Collagen Secretion in Diseased Human Patient-Derived Precision-Cut Liver Slices (hPCLS)

**Figure 1:**
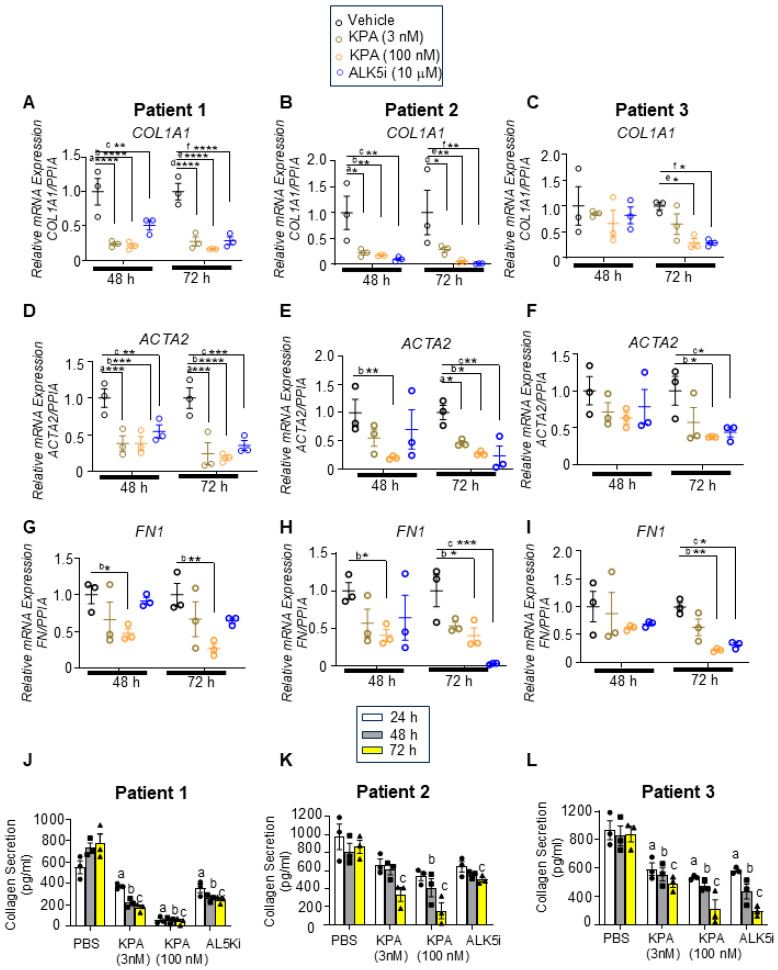
KPA reduces fibrogenic gene expression as well as type 1 collagen secretion in human fibrotic precision-cut liver slices (PCLSs). Human PCLSs generated from patients with fibrotic livers were treated with KPA (3 nM, 100 nM), vehicle (PBS), or TGFβ receptor 1 kinase inhibitor II (ALK5i, 10 mM) for 48 or 72 h. (**A**–**I**) Changes in fibrogenic gene expression determined by qPCR. a, *p* < 0.05 vs. PBS (vehicle); b, *p* < 0.05 vs. PBS (vehicle); and c, *p* < 0.05 vs. PBS (vehicle) for each time point. (**J**–**L**) Secreted pro-collagen I alpha 1 protein in culture media measured by ELISA. a, *p* < 0.05 vs. PBS (vehicle) secretion (24 h); b, *p* < 0.05 vs. PBS (vehicle) secretion (48 h); and c, *p* < 0.05 vs. PBS (vehicle) secretion (72 h). For (**A**–**I**) (data are shown from N = 3 patients), results are expressed as mean +/− S.E.M. * *p* < 0.05 vs. control. ** *p* < 0.01 vs. control. *** *p* < 0.001 vs. control. **** *p* < 0.0001 vs. control. Two-way ANOVA was used, followed by multiple comparison test.

To test our hypothesis that kisspeptin has an anti-fibrotic effect in the human liver, we utilized hPCLS generated from stromal (*fibrotic, non-tumor)* biopsies from three male patients with HCC (Table 1). Liver slices were treated with the potent kisspeptin analog, TAK-448 (3 nM, 100 nM, henceforth referred to as KPA) for 48 h or 72 h. These KPA concentrations were selected since they decreased triglyceride accumulation and increased mitochondrial b-oxidation in primary mouse hepatocytes, and stimulated insulin secretion in isolated human pancreatic islets [13,25]. Since the TGFβ receptor 1 kinase inhibitor II (ALK5i) exhibits anti-fibrogenic effects by targeting TGFβ signaling [21], we used ALK5i as a positive control in these studies. There was no impact of KPA or ALK5i on the viability of hPCLSs (Figure S1). KPA treatment significantly lowered the mRNA levels of key fibrogenic genes *COL1A1* (encodes collagen)*, ACTA2* (encodes a-smooth muscle actin, α-SMA), and *FN1* (encodes fibronectin) compared to the control (PBS) in each patient (Figure 1A–I). A similar effect was observed with ALK5i. KPA treatment also significantly reduced collagen secretion by hPCLS (Figure 1 J–L), which is consistent with the effects on the level of *COL1A1* transcript (Figure 1 A–C). 

**Figure 2:**
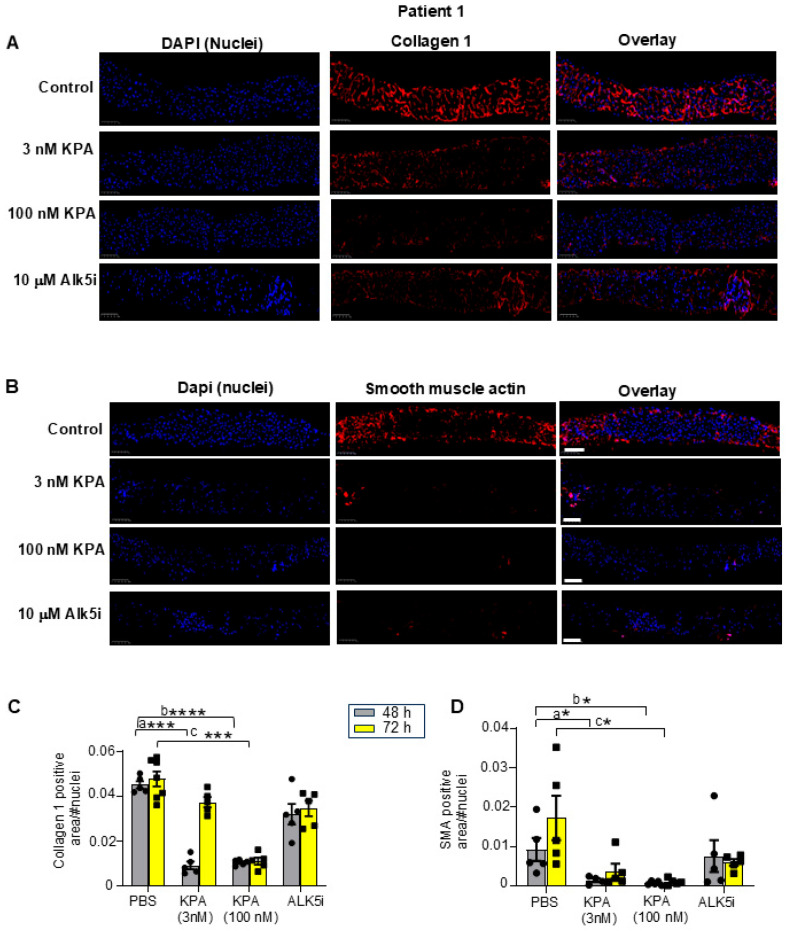
KPA treatment reduces collagen and smooth muscle actin (SMA) expression in hPCLS. Representative images of human PCLS generated from fibrotic liver biopsy from Patient 1, treated with KPA, vehicle (PBS), or ALK5i for 48 h and immunostained for (**A**) collagen 1 and (**B**) smooth muscle actin. Scale bars: 250 mm. (**C**,**D**) Quantification of collagen and smooth muscle actin (SMA) immunostaining in hPLCS from Patient 1 treated with KPA (3 nM, 100 nM), vehicle (PBS), or ALK5i (10 mM) for 48 h or 72 h (4 technical replicates). See Figure S2 for additional images from Patients 2 and 3. Results: mean +/− S.E.M. a, *p* < 0.05 vs. PBS (48 h) for KPA 3 nM; b, *p* < 0.05 vs. PBS (48 h) for KPA 100 nM; c, *p* < 0.05 vs. PBS (vehicle, 72 h), * *p* < 0.05 vs. control. *** *p* < 0.001 vs. control. **** *p* < 0.0001 vs. control. Two-way ANOVA was used, followed by multiple comparison test.

To detect changes in fibrogenic protein expression following KPA treatment, immunohistochemical analysis was conducted. The results revealed that after 48 h and 72 h of KPA (3 nM, 100 nM) treatment, collagen 1 and a-SMA protein expression was reduced compared to untreated liver slices (Figure 2A–D, Figures S2 and S3). These observations were similar to the effect of ALK5i treatment (Figure 2A–D, Figures S2 and S3). The evaluation of representative H&E-stained liver sections showed that 48 h of KPA treatment did not alter tissue architecture (Figure S4). Picrosirius red staining revealed the extent of fibrosis in each patient’s liver prior to any treatment as measured blindly by a pathologist, revealing F1, F2, and F4 stages of fibrosis for patients 1, 2, and 3, respectively (Figure S4 and Table 1). 

**Figure 3:**
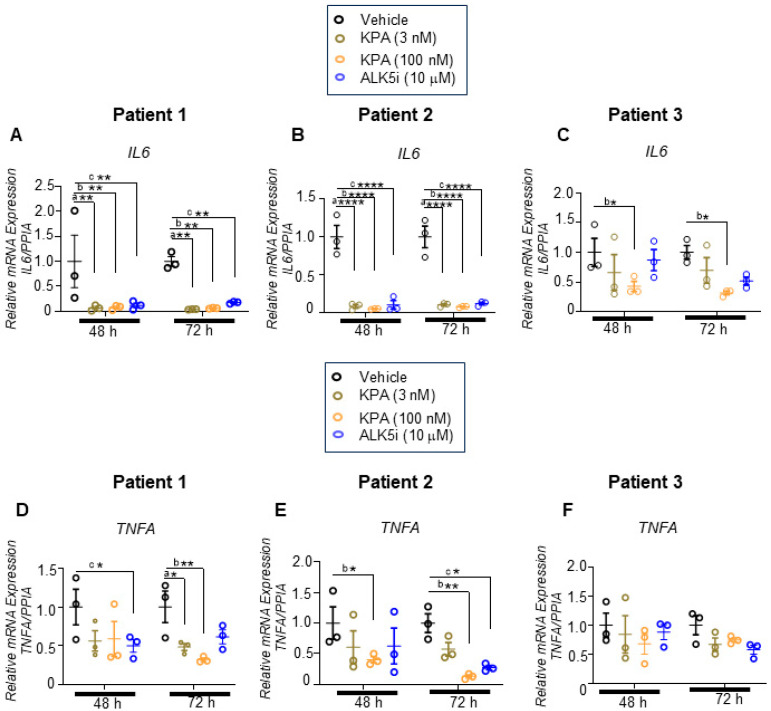
KPA reduces inflammatory gene expression in hPCLS. Human PCLSs generated from patients with fibrotic livers were treated with KPA (3 nM, 100 nM), vehicle (PBS), or ALK5i (10 mM) for 48 or 72 h. Changes in gene expression were determined by qPCR for (**A**,**D**) Patient 1, (**B**,**E**) Patient 2, and (**C**,**F**) Patient 3. Data are shown from *n* = 3 patients. a, *p* < 0.05 vs. PBS (vehicle); b, *p* < 0.05 vs. PBS (vehicle); and c, *p* < 0.05 vs. PBS (vehicle) for each time point. Results are expressed as mean +/− S.E.M. * *p* < 0.05 vs. control. ** *p* < 0.01 vs. control. **** *p* < 0.0001 vs. control. Two-way ANOVA was conducted, followed by multiple comparison test.

Having observed that KPA reduced fibrogenesis, we assessed the effect of 48 h and 72 h of KPA treatment on inflammatory markers. In both cases, KPA treatment robustly reduced the expression of interleukin-6 (*IL-6*) mRNA levels at 48 h, which was sustained at 72 h (Figure 3A–C) without any impact on drug toxicity (Figure S1). Similar effects were observed with ALK5i (Figure 3A–C; Figure S1). For tumor necrosis factor-α (*TNFA),* KPA only decreased *TNFA* mRNA levels at the higher dose in Patients 1 and 2, with the effect being more pronounced after 72 h (Figure 3D–F). In patient 3, *TNFA* mRNA expression decreased in response to KPA (3 nM and 100 nM) and ALK5i treatment, especially after 72 h, although statistical significance was not achieved. Taken together, these results reveal for the first time that KPA displays anti-fibrotic and anti-inflammatory properties in human fibrotic liver samples.

### 3.2. KPA Treatment Upregulates KISS1 and KISS1R in hPCLS, and KISS1R Is Strongly Expressed in Human HSCs

**Figure 4:**
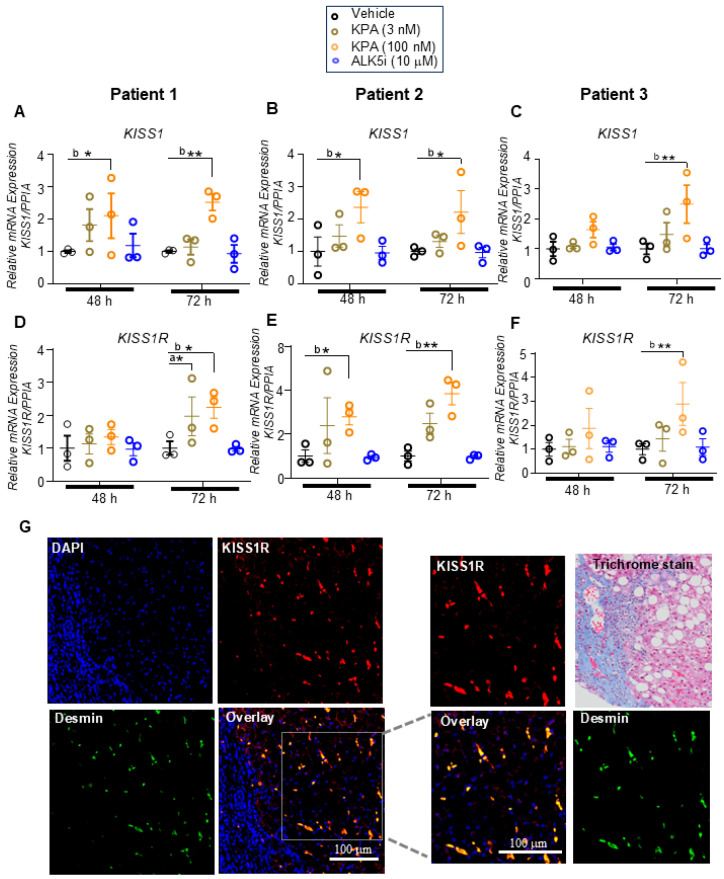
KPA treatment upregulates *KISS1* and *KISS1R* expression in hPCLS. Human PCLS generated from patients with fibrotic liver biopsies were treated with KPA (3 nM, 100 nM), vehicle (PBS), or ALK5i (10 mM) for 48 or 72 h. Changes in (**A**–**C**) *KISS1* and *KISS1R* (**D**–**F**) gene expression were determined by qPCR in patient livers. Data are shown from *n* = 3 patients. Results are expressed as mean +/− S.E.M. * *p* < 0.05 vs. control. ** *p* < 0.01 vs. control. a, *p* < 0.05 vs. PBS (vehicle); b, *p* < 0.05 vs. PBS (vehicle); and c, *p* < 0.05 vs. PBS (vehicle), for each time point. Two-way ANOVA was conducted, followed by multiple comparison test. (**G**) Representative confocal images of human hepatic stellate cells in MASH patient biopsies, immunostained for endogenous KISS1R (red) and desmin (green), a marker for stellate cells. Areas of colocalization (yellow) are shown in overlay; magnified images and trichrome staining of liver fibrotic section are shown on the right.

To further investigate the mechanistic actions of KPA in human liver slices, we examined the expression of *KISS1* and *KISS1R* mRNA levels and observed that 100 nM KPA significantly induced *KISS1* and *KISS1R* mRNA levels after 72 h of treatment (Figure 4A–F). In contrast, ALK5i treatment had no effect on *KISS1* and *KISS1R* transcript levels (Figure 4A–F). Additionally, using validated antibodies [13,29,30], immunohistochemical analysis of endogenous KISS1R in human livers from MASH patients with fibrosis revealed that KISS1R is robustly expressed in hepatic stellate cells (red) and colocalizes (yellow, overlay) with desmin (green), an intermediate filament marker of hepatic stellate cells [31] (Figure 4G). Taken together, these results led us to conclude that the activation of KISS1R has anti-fibrogenic roles, and one potential mechanism by which KPA exerts a protective role is via the upregulation of *KISS1* and *KISS1R* levels in patient livers. 

### 3.3. KPA Treatment Attenuates Fibrogenesis in Hepatic Stellate Cells (HSCs)

**Figure 5:**
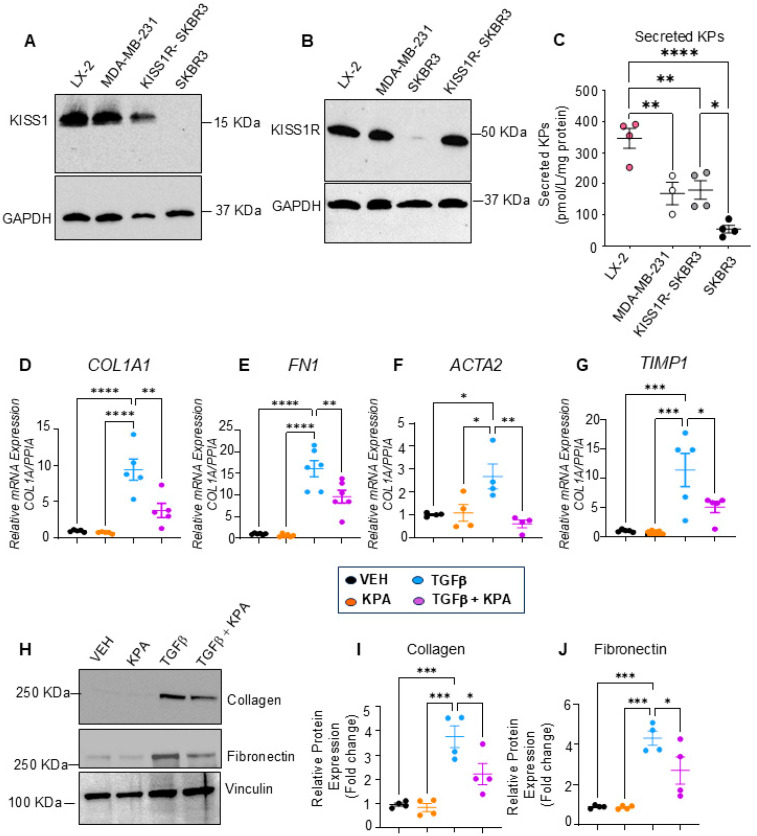
KPA treatment decreases activation of human hepatic stellate LX-2 cells. Representative Western blots showing expression of endogenous (**A**) KISS1 protein (*n* = 5 biological replicates) and (**B**) KISS1R protein expression (*n* = 4 biological replicates). MDA-MB-231, SKBR3, and KISS1R-SKBR3 were used as reference for expression (*n* = 4). (**C**) Secreted kisspeptin protein in culture media measured by ELISA (N-4 biological replicates). (**D**–**G**) Changes in fibrogenic gene expression in response to KPA (3 nM, 48 h) +/− TGFb (5 ng/mL, 48 h). (*n* = 4–6 biological replicates) (**H**) Western blot analysis and (**I**,**J**) densitometric analyses of blots, showing changes in fibrogenic protein in response to KPA (3 nM, 72 h) +/− TGFb (5 ng/mL, 72 h). (*n* = 4 biological replicates) Results are expressed as mean +/− S.E.M. * *p* < 0.05 vs. control. ** *p* < 0.01 vs. control. *** *p* < 0.001 vs. control. **** *p* < 0.0001 vs. control. One-way ANOVA was conducted, followed by multiple comparison test.

To better understand the anti-fibrotic roles of KISS1R signaling in the liver, we used the immortalized human hepatic stellate cell line (LX-2), which is a highly established cellular model of human hepatic fibrogenesis [24]. To initiate these studies, we first quantified KISS1 and KISS1R protein levels in LX-2 cells. We used human MDA-MB-231 and SKBR3 breast cancer cell lines as positive controls to analyze KISS1 and KISS1R protein levels. We have previously shown that MDA-MB-231 cells express high endogenous levels of KISS1 and KISS1R [28,29,30]. In contrast, native (parental) SKBR3 cells do not, but when KISS1R is stably expressed in these cells (KISS1R-SKBR3), it leads to the robust expression of KISS1R, as well as KISS1 and kisspeptin secretion [28,30]. We observed that LX-2 cells express KISS1 and KISS1R proteins endogenously at levels similar to MDA-MB-231 and KISS1R-SKBR3 cells (Figure 5A,B). Our results also showed that LX-2 cells secrete KPs, and interestingly, this occurred to a greater extent than MDA-MB-231 or KISS1R-SKBR3 cells (Figure 5C). 

**Figure 6:**
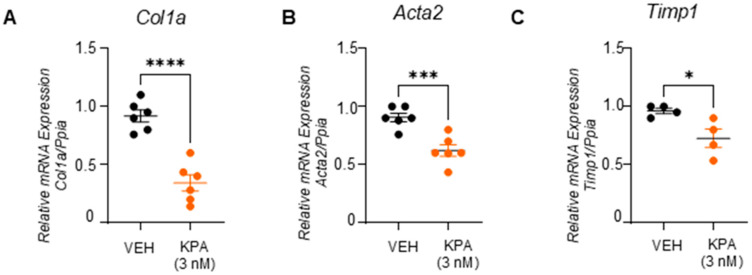
KPA reduces fibrogenic markers in primary mouse HSCs. Gene expression by qPCR analysis showing the effect of KPA (3 nM, 48 h) treatment on (**A**) *Col1a*, (**B**) *Acta2,* and (**C**) *Timp1.* (*n* = 4–6 biological replicates) Results are expressed as mean +/− S.E.M. Student’s unpaired *t*-test. * *p* < 0.05 vs. control. *** *p* < 0.001 vs. control. **** *p* < 0.0001 vs. control.

It was reported that, in response to potent profibrogenic factors such as TGFβ-activated hepatic stellate cells in the injured liver, fibrosis is promoted by upregulating extracellular proteins such as collagen, smooth muscle actin, and fibronectin and tissue inhibitors of metalloproteinases 1 (TIMP-1) [32]. We, therefore, used this cell model to study the effect of KPA on fibrogenesis. Serum-starved LX-2 cells treated with TGFβ (5 ng/mL) for 48 h upregulated both the mRNA (*COL1A1, FN, ACTA2,* and *TIMP1*) and protein (collagen and fibronectin) levels of fibrogenic markers, and this upregulation was significantly blocked in the presence of KPA (3 nM) (Figure 5D–J). Furthermore, in primary mouse hepatic stellate cells, KPA (3 nM) treatment for 48 h significantly reduced the mRNA expression of profibrogenic markers, *Col1a1,* and *Acta2*, and lowered the expression of *Timp1* (Figure 6).

**Figure 7:**
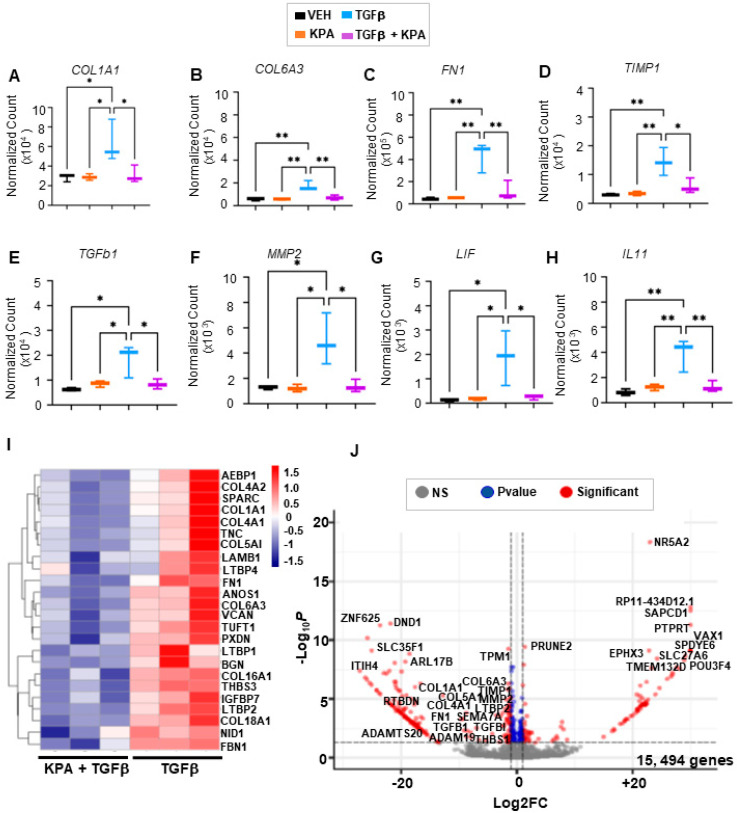
Effect of KPA treatment on gene expression in human hepatic stellate cells by RNA-seq. RNA was extracted from LX-2 cells treated with PBS (vehicle), KPA (3 nM, 48 h), or +/− TGFβ (5 ng/mL, 48 h). Changes in fibrogenic (**A**–**F**) and inflammatory (**G**,**H**) genes were identified by transcriptomic analysis. Data are presented as (**I**) heatmap and (**J**) volcano plot of differentially expressed genes (padj < 0.1) in LX-2 cells treated with KPA (3 nM, 48 h) + TGFβ (5 ng/mL, 48 h) compared to TGFβ alone. (*n* = 3 biological replicates). Results are expressed as mean +/− S.E.M. * *p* < 0.05 vs. control. ** *p* < 0.01 vs. control. One-way ANOVA was performed, followed by multiple comparison test.

**Figure 8:**
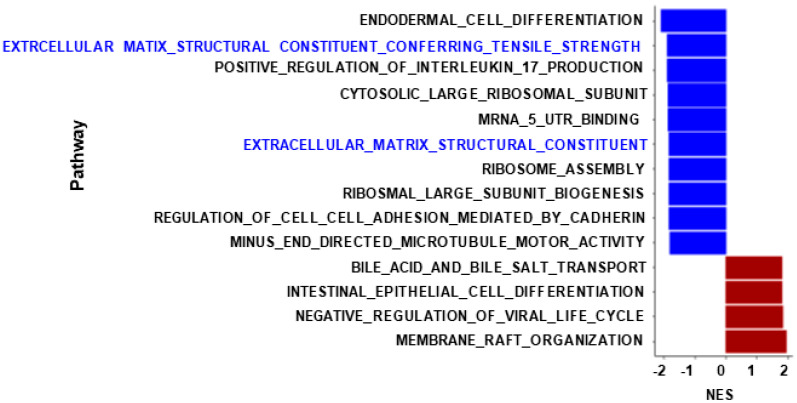
KPA downregulates molecular pathways related to extracellular matrix production in HSCs. Gene set enrichment analysis of LX-2 cells after 48 h of treatment with KPA (3 nM), TGFβ (5 ng/mL), or TGFβ alone is shown. X-axis represents normalized enrichment scores of gene sets (blue: downregulated pathways; red: upregulated pathways).

Changes in profibrogenic markers in LX-2 cells were confirmed in the RNA-seq datasets after 48 h of treatment with KPA (3 nM) in combination with TGFb (5 ng/mL), in contrast to cells treated with TGFβ (5 ng/mL) alone (Figure 7A–F). Gene enrichment analysis revealed that pathways regulating the extracellular matrix were downregulated in KPA-treated groups in the presence of TGFβ in contrast to TGFβ alone (Figure 8). KPA treatment also significantly decreased TGFβ-induced increases in leukemia inhibitory factor (*LIF*) and interleukin 11 (*IL11*), two members of the IL6 family of cytokines (Figure 7G,H). Co-treatment with KPA also decreased the expression of genes (Figure 7I,J), regulating the progression of fibrosis (e.g., *COL6A3*, *AEBP1*, *SPARC*, *TNC*, *LAMB1*, *BGN*) and hepatocellular carcinoma (e.g., *COL4A1, COL4A2, TUFT1*, *VCAN, NID1*).

**Figure 9:**
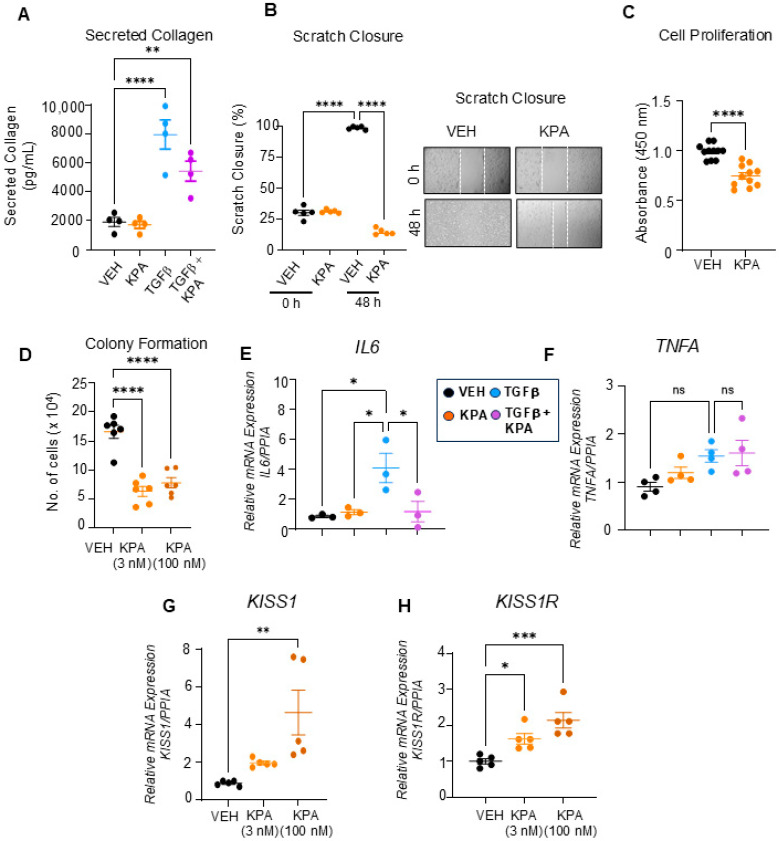
KPA treatment decreases collagen secretion, cell migration, and proliferation of LX-2 cells. Quantification of (**A**) TGFβ-induced collagen secretion following VEH, KPA (3 nM), TGFβ (5 ng/mL), and KPA + TGFβ treatment (*n* = 4 biological replicates); (**B**) cell migration following VEH and KPA (3 nM) treatment. KPA treatment decreases (*n* = 4 biological replicates) (**C**) LX-2 cell proliferation following VEH and KPA (3 nM) treatment, as assessed using BrdU (*n* = 11 biological replicates) and (**D**) soft agar colony formation following VEH, KPA (3 nM, 100 nM) treatment (*n* = 11 biological replicates) t. Gene expression by qPCR analysis showing the effect of KPA (3 nM, 48 h) treatment on inflammatory markers (**E**,**F**) *IL6* and *TNFA*, and (**G**,**H**) *KISS1* and *KISS1R* (*n* = 3–5 biological replicates). Results are expressed as mean +/− S.E.M. Student’s unpaired *t*-test or one-way ANOVA was performed, followed by multiple comparison test. * *p* < 0.05 vs. control. ** *p* < 0.01 vs. control. *** *p* < 0.001 vs. control. **** *p* < 0.0001 vs. control.

Activated HSCs secrete collagen, which, upon deposition, leads to fibrosis. We, therefore, examined the effect of KPA on TGFβ-induced collagen secretion and found that it was diminished by KPA treatment (3 nM) (Figure 9A). To further understand the cellular action of KPA on LX-2 cells, we conducted cell migration, proliferation, and colony formation assays. We found that KPA reduced LX-2 cell migration, as assessed using the scratch/wound healing assay (Figure 9B), and decreased cell proliferation, as examined by the BrdU assay (Figure 9C). It was also observed that KPA (3 nM, 100 nM) decreased LX-2 colony formation on soft agar (Figure 9D), suggesting that KPA inhibits cell transformation. The effect of KPA on the expression of inflammatory markers was also assessed. KPA treatment reduced TGFβ-induced *IL6* expression (Figure 9E) but did not significantly change TGFβ-induced *TNFA* mRNA levels (Figure 9F). Similar to the observations with hPCLS, KPA (100 nM) significantly upregulated *KISS1* and *KISS1R* mRNA levels (Figure 9G,H). Taken together, these findings suggest that KPA directly attenuates hepatic fibrogenesis by decreasing stellate cell activation.

### 3.4. KPA Inhibits TGFβ Signaling in Hepatic Stellate Cells via Activation of Protein Phosphatase PP2A

TGFβ is a major profibrogenic cytokine that plays a significant role in hepatic fibrogenesis since it potently induces hepatic stellate cell activation [20,33]. In canonical TGFβ signaling, TGFβ binds to the TGFβ type II receptor (TGFβRII) and stimulates the recruitment of the TGFβ type I receptor (TGFβRI). TGFβRI then binds and phosphorylates its substrates, which are receptor-activated SMADs, specifically SMAD2 and SMAD3. These phosphorylated SMADs then form a heterocomplex with the shared partner, SMAD4. This complex translocates to the nucleus to activate the transcription of multiple target profibrogenic genes, such as *COL1A1, ACTA2, FN, TIMP-1,* and *SNAI1* [20,34,35].

**Figure 10:**
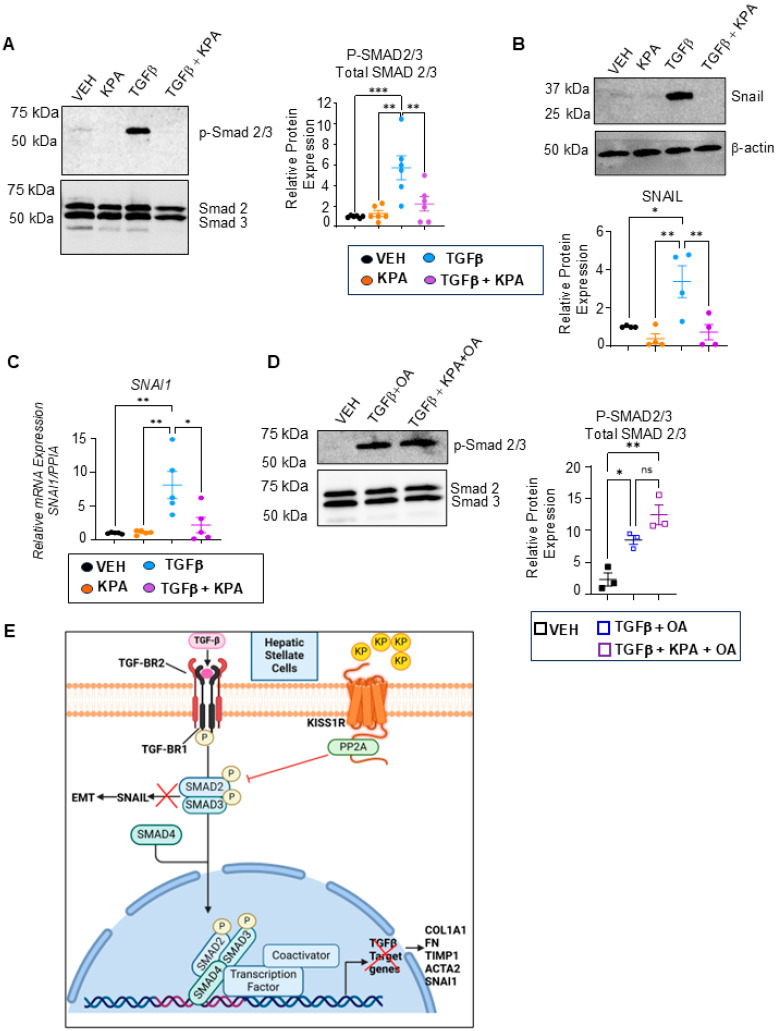
KPA treatment decreases TGFβ signaling in human HSCs. (**A**) TGFβ-induced SMAD phosphorylation (*n* = 5 biological replicates) and (**B**) SNAIL expression (*n* = 4 biological replicates) was observed, following KPA (3 nM, 72 h) treatment, as determined by Western blot analysis; densitometric analysis of blots are shown below. (**C**) TGFβ-induced *SNAI1* mRNA expression following KPA (3 nM, 48 h) determined by qPCR (*n* = 4 biological replicates). (**D**) TGFβ-induced SMAD 2/3 phosphorylation cells were pretreated with OA (5 nM) for 3 h, following TGFβ1 (5 ng/mL), and/or KPA (3 nM) for 48 as determined by Western blot analysis. OA treatment did not impact cell viability (see Figure S5) (*n* = 4 biological replicates). (**E**) Schematic showing proposed signaling pathways by which KISS1R activation with kisspeptin (KP) suppresses hepatic fibrosis in hepatic stellate cells. Results are expressed as mean +/− S.E.M. * *p* < 0.05 vs. control. ** *p* < 0.01 vs. control. *** *p* < 0.001 vs. control. One-way ANOVA was performed, followed by multiple comparison test.

To investigate the potential mechanism by which KISS1R signaling improves hepatic fibrogenesis, the effect of KPA on TGFβ1/SMAD signaling was examined in LX-2 cells. Treatment with TGBβ induced the phosphorylation of *p*-SMAD2/3, which was significantly reduced in the presence of KPA (Figure 10A). TGBβ treatment also resulted in the robust expression of its downstream target, SNAIL, a mesenchymal marker that is a critical regulator of hepatic stellate cell activation [35]. KPA treatment attenuated the TGBβ-induced expression of SNAIL mRNA and the protein (Figure 10B,C). The KISS1R C-terminal directly binds serine/threonine protein phosphatase type 2A (PP2A) [36]. The activation of PP2A promotes the dephosphorylation of SMAD 2/3 [37]. We, therefore, investigated whether the pharmacological inhibition of PP2A using okadaic acid (OA) in the presence of kisspeptin interferes with TGBβ-SMAD signaling. Cells were pretreated with OA (5 nM) for 3 h before introducing TGFβ1 (5 ng) and KPA (3 nM) to study SMAD2/3 phosphorylation after 48 h; OA treatment did not impact cell viability (Figure S5). Western blot analysis revealed that treatment with a protein phosphatase inhibitor abolished the effects of KPA on the dephosphorylation of SMAD2/3 (Figure 10D).

Overall, the present data indicate how KPA/KISS1R signaling directly suppresses hepatic fibrogenesis by inhibiting TGBβ signaling through regulating PP2A phosphatases (Figure 10E), thus identifying it as a new therapeutic target to potentially treat fibrosis.

## 4. Discussion

Kisspeptin is well studied as a neuropeptide that critically regulates pubertal and reproductive development by acting on the hypothalamus to stimulate gonadotropin-releasing hormone secretion and the release of gonadotropins (follicle-stimulating and luteinizing hormones) from the pituitary. Hypothalamic KISS1 and KISS1R expressions decrease in many pathological conditions, resulting in the delay or absence of puberty in children, hypogonadotropic hypogonadism, and reproductive disorders [8,38]. The chronic administration of kisspeptin is safe and can restore normal pubertal development in children with delayed puberty [20] in addition to treating hypothalamic amenorrhea in women and hyposexual drive disorder in otherwise healthy males [39]. 

Outside the brain, kisspeptin and its receptor are expressed in tissues such as the liver, gonads, uterus, placenta, small intestine, and adrenal glands [8,40]. KISS1 and KISS1R are also expressed in pancreatic islet a and b cells [41,42]. The administration of kisspeptin to human, mouse, rat, and pig-isolated pancreatic b cells directly increased glucose-stimulated insulin secretion (GSIS) [42]. The acute peripheral administration of kisspeptin in humans, mice, rats, and monkeys in vivo also resulted in increased GSIS [25,39]. These studies demonstrate an important role for kisspeptin signaling in regulating glucose homeostasis. Hepatocytes express kisspeptin and KISS1R [13]. In the liver, the deletion of *Kiss1r* in hepatocytes augments glucose intolerance and insulin resistance in an HFD-fed mouse model of MASLD [13], in addition to increasing hepatic steatosis and its progression to MASH [13]. In contrast, the treatment of HFD-fed male C57BL6/J mice with KPA for 6 weeks improved insulin sensitivity and glucose tolerance and ameliorated steatosis. To evaluate the impact of KPA administration on advanced MASH and fibrosis, male DIAMOND mice were utilized [13]. These mice, when maintained on HFD and a sugar/water diet, recapitulate human MASLD by developing obesity, insulin resistance, fatty liver, steatohepatitis, fibrosis, and HCC [18]. It was observed that 6 weeks of KPA administration to these DIAMOND mice improved advanced fibrosis without a significant change in body weight. Thus, the effect of KPA administration was not due to changes in body weight [13]. Mechanistically, it was observed that hepatic KISS1R signaling decreases hepatic lipogenesis and MASH progression by activating the master energy sensor, AMP-activated protein kinase (AMPK), and increasing mitochondrial fatty acid oxidation. It was also observed that KPA-treated DIAMOND mice livers displayed lower mRNA and protein levels of fibrogenic markers (e.g., TGFβ, SMA, collagen, matrix metalloproteinases (MMP), such as MMP-2, MMP-9 and MMP-13, and decreased levels of inflammatory markers (e.g., interleukin 1b), and diminished NFk-b signaling [13]. However, whether kisspeptin had any impact on regulating human hepatic fibrosis is unknown. 

In this study, we provide strong evidence that KPA exhibits anti-fibrotic activity, using human precision-cut ultrathin liver slices and a relevant ex vivo human model of fibrotic liver. HPCLS are a powerful three-dimensional model that maintains the complex organization, cellular heterogeneity, and micro-environment of the liver, as well as the pathological characteristics of human disease [43]. Fibrotic liver sections obtained from patients with HCC were utilized for this study, as verified by a pathologist (tumor sections were excluded). Male patients were included in this initial study, as MASLD is more prevalent in males than in females [44]. We demonstrate for the first time that KISS1R is strongly expressed in human hepatic stellate cells in fibrotic MASH patient liver biopsies. KISS1 and KISS1R are also expressed in hepatocytes, and the liver produces kisspeptin [13,14]. Thus, attenuating signals from KISS1R expressed in other cell types, such as hepatocytes, may contribute to the anti-fibrogenic effects of kisspeptin signaling. 

KPA, created by the modification of kisspeptin-10 (a naturally occurring kisspeptin peptide), exhibits increased stability and potency [45]. As reported here, KPA lowered the expression of fibrogenic markers (*COL1A, FN1, ACTA2*) in hPCLS, decreased collagen secretion, and reduced the expression of inflammatory markers (*IL-6, TNFA*). This effect was similar to TGFβ receptor 1 kinase inhibitor II (ALK5i), which has been shown to have a beneficial effect on MASLD [21,46]. These results are consistent with our observations made in the DIAMOND mouse model [13], suggesting the anti-fibrogenic potential of KPA in humans with fibrotic liver disease.

Another major finding from this study was made using human HSCs and primary mouse HSCs, where we showed for the first time that KPA decreases fibrogenesis through the downregulation of the canonical TGFβ signaling pathway. This leads to the decreased expression of TGFβ-target genes *(COL1A, FN-1, TIMP-1, SNAI1)* that are implicated in fibrosis. TGFβ plays a key role in the progression of liver fibrosis, and drugs that inhibit TGFβ have anti-fibrotic effects [20]. We showed that, in stellate cells, KPA specifically decreases TGFβ-induced p-SMAD2/3 levels. SMAD2/3 has been shown to mediate extracellular matrix accumulation and fibrosis in the liver [33,47] and to regulate epithelial–mesenchymal transition (EMT) and metastasis in response to TGFβ in HCC [48]. We also showed that in HSCs, KPA treatment reduces the expression of the profibrogenic, EMT-related transcription factor, SNAIL, which is a TGFβ-target gene [49,50]. Our data suggest that KPA’s effect on promoting the dephosphorylation of phospho-SMAD2/3 in the presence of TGFβ1 is mediated, at least in part, by increasing the activity of protein phosphatase PP2A, which is a direct binding partner of KISS1R. Together, these data provide evidence for the involvement of the TGFβ1/SMAD2/3 pathway in the kisspeptin-mediated inactivation of HSC.

Another interesting finding resulting from this study was the observation that KPA upregulates KISS1/KISS1R expression in hPCLS and LX-2, suggesting another mechanism of action by which KPA mediates its protective effect. This observation is also supported by our current and previous findings that the overexpression of KISS1R in SKBR3 cells result not only in increased levels of KISS1R but also those of KISS1 [28]; this has also been reported to occur in human endometrial cells upon the exogenous expression of KISS1R [51]. These findings suggest that there is a positive feedback loop between KISS1 and KISS1R. The expressions of KISS1 and KISS1R mRNA and protein are low in healthy human liver but increase in liver biopsies from MASH patients compared with those of healthy subjects [13]. Plasma kisspeptin levels are also increased in patients with steatosis and MASH, compared to healthy subjects, suggesting that the upregulation of the KISS1R signaling pathway is a compensatory and protective response aiming to resolve MASH [13]. In further support of this idea, studies in mice showed that liver *Kiss1* expression increases in genetic models of obesity and type 2 diabetes (db/db and ob/ob mice) [14]. We and others have also observed the increased expression of *Kiss1* and *Kiss1r* in a high-fat diet-fed mouse model of MASH [13,14,52]. Thus, the upregulation of hepatic KISS1/KISS1R by KPA may serve to slow down or reverse disease progression. Future studies will investigate the mechanism(s) by which KPA treatment leads to an upregulation of KISS1/KISS1R.

Earlier this year, the thyroid hormone receptor β agonist Resmetirom was the first drug approved by the FDA to treat MASH, having shown improved key readouts of liver pathology in 25–30% of patients in a phase III clinical trial [53]. This highlights the continuous need for new therapeutics. Our translational observations provide first-time evidence for the therapeutic potential of KP peptides in a human setting of fibrosis and support the use of KISS1R-activating strategies in clinical trials.

## Figures and Tables

**Table 1:** Characteristics of human liver donors used for hPCLS studies.

Patient ID	Age (Years)	Sex	Disease Background	Pathological Diagnosis	Fibrosis Stage
1	76	Male	Patient has underlying HBV diagnosed with a mass in segment 5/6 of the liver (consistent with HCC)	Mild portal and mild interface hepatitis, compatible with the patient’s history of hepatitis B	F1
2	66	Male	HCC (HBV-infected chronic hepatitis)	Mild portal chronic inflammation and periportal fibrosis with bridging fibrous septa. No interface hepatitis or lobular inflammation and steatosis.	F2
3	53	Male	HCC (HCV-infected chronic hepatitis)	Nodular liver parenchyma without significant portal or lobular inflammation. No significant steatosis.	F4

## Data Availability

The RNA-seq dataset was deposited to GEO (Gene Expression Omnibus). It will be made publicly available once this manuscript is published.

## Supplementary Materials

The following supporting information can be downloaded at: https://www.mdpi.com/article/10.3390/cells13191651/s1, Figure S1–S5: Supplementary Figures and blots; Table S1: Primers.
