# Supplementary material for: Kisspeptin Alleviates Human Hepatic Fibrogenesis by Inhibiting TGFβ Signaling in Hepatic Stellate Cells
Source: Cells. 2024 Oct 4;13(19):1651. doi: 10.3390/cells13191651 (PMC11476267; doi:10.3390/cells13191651)
Supplement: Supplementary file 1 [file cells-13-01651-s001.zip › Table S1 primers.pdf]

Table S1: Primers

**Name**	**Forward**	**Reverse**
**Mouse**		
Acta2	ACTGGGACGACATGGAAAAG	GTTCAGTGGTGCCTCTGTCA
Col1a1	GAGAGAGCATGACCGATGGATT	TGTAGGCTACGCTGTTCTTGCA
Timp1	CCTTGCAAACTGGAGAGTGACA	AGGCAAAGTGATCGCTCTGGT
Fn1	CGAGGTGACAGAGACCACAA	CTGGAGTCAAGCCAGACACA
Rpl13a	GCTGCTCTCAAGGTTGTTCG	CCTTTTCCTTCCGTTTCTCC
**Human**		
ACTA2	CATCCTCATCCTCCCTTGAG	ATGAAGGATGGCTGGAACAG
COL1A1	CCGGCTCCTGCTCCTCTTAGCG	CGTTCTGTACGCAGGTGATTGGTGG
TIMP1	CAAGATGTATAAAGGGTTCCAAGC	TCCATCCTGCAGTTTTCCAG
FN1	AAACCAATTCTTGGAGCAGG	CCATAAAGGGCAACCAAGAG
IL6	CATCCTCGACGGCATCTCAG	GCTCTGTTGCCTGGTCCTC
TNFA	GAGGCCAAGCCCTGGTATG	CGGGCCGATTGATCTCAGC
KISS1	GGACCTGCCTCTTCTCACCA	ATTCTAGCTGCTGGCCTGTG
KISS1R	CCCACCCTCTGGACATTCAC	CCTAGAAGTGCCTTGAGGCTTG
SNAI1	TCGGAAGCCTAACTACAGCGA	AGATGAGCATTGGCAGCGAG
PPIA	CCCACCGTGTTCTTCGACATT	GGACCCGTATGCTTTAGGATGA
