# Supplementary material for: Kisspeptin Alleviates Human Hepatic Fibrogenesis by Inhibiting TGFβ Signaling in Hepatic Stellate Cells
Source: Cells. 2024 Oct 4;13(19):1651. doi: 10.3390/cells13191651 (PMC11476267; doi:10.3390/cells13191651)

Prasad et al., BLOTS

FIGURE 5A

KISS1 expression in human hepatic stellate cell (LX-2)

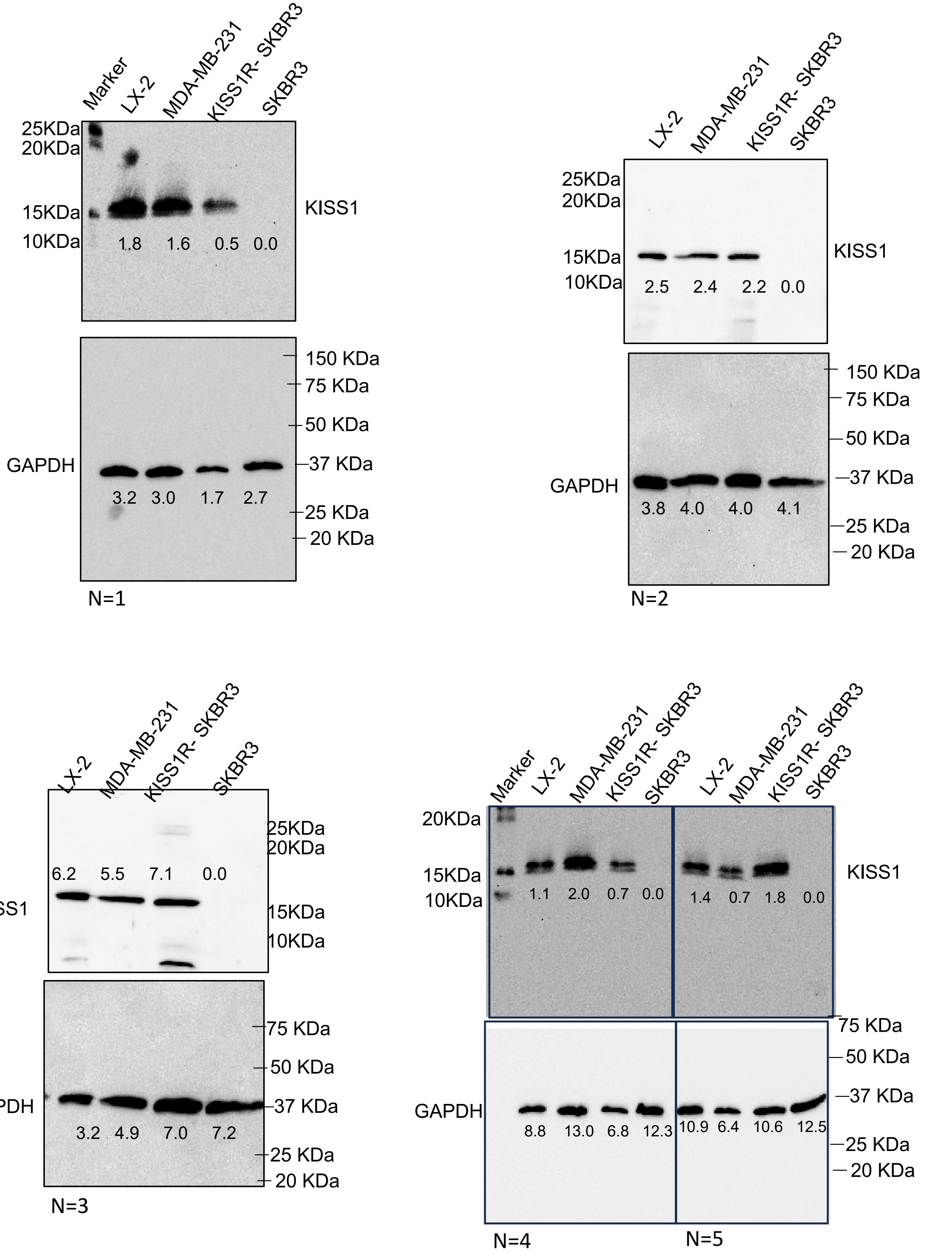

Prasad et al., BLOTS

FIGURE 5B

KISS1R expression in human hepatic stellate cell (LX-2)

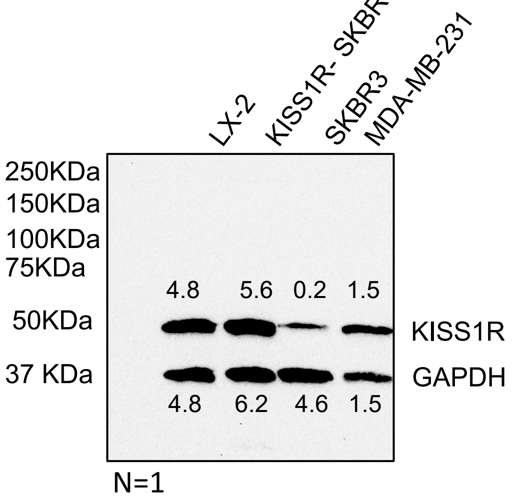

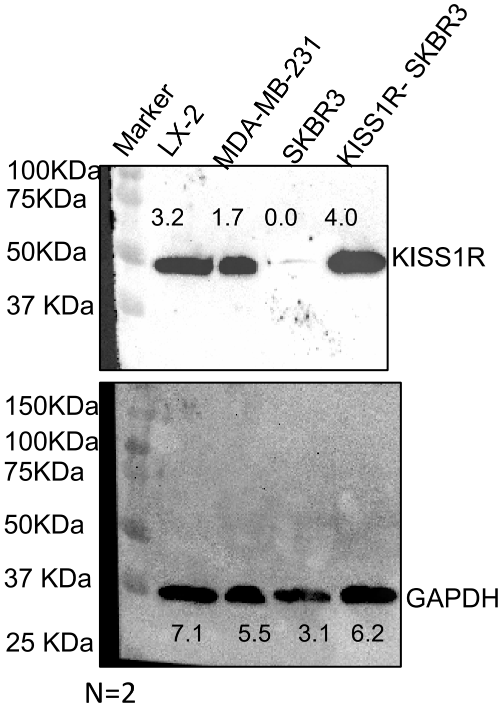

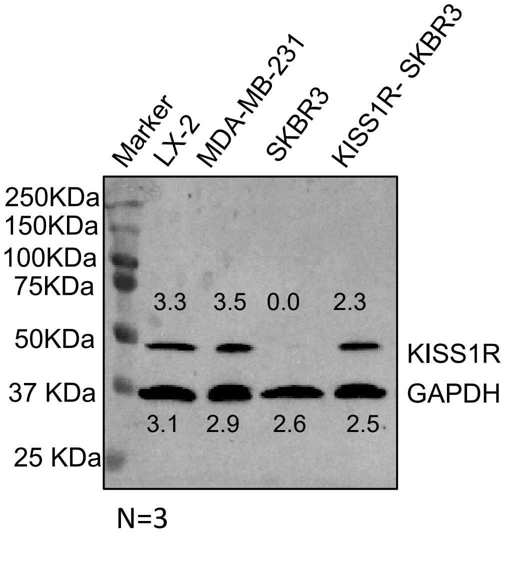

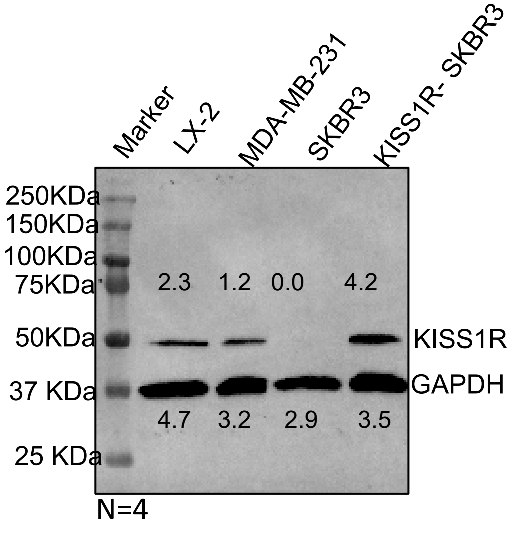

Prasad et al., BLOTS

FIGURE 5H

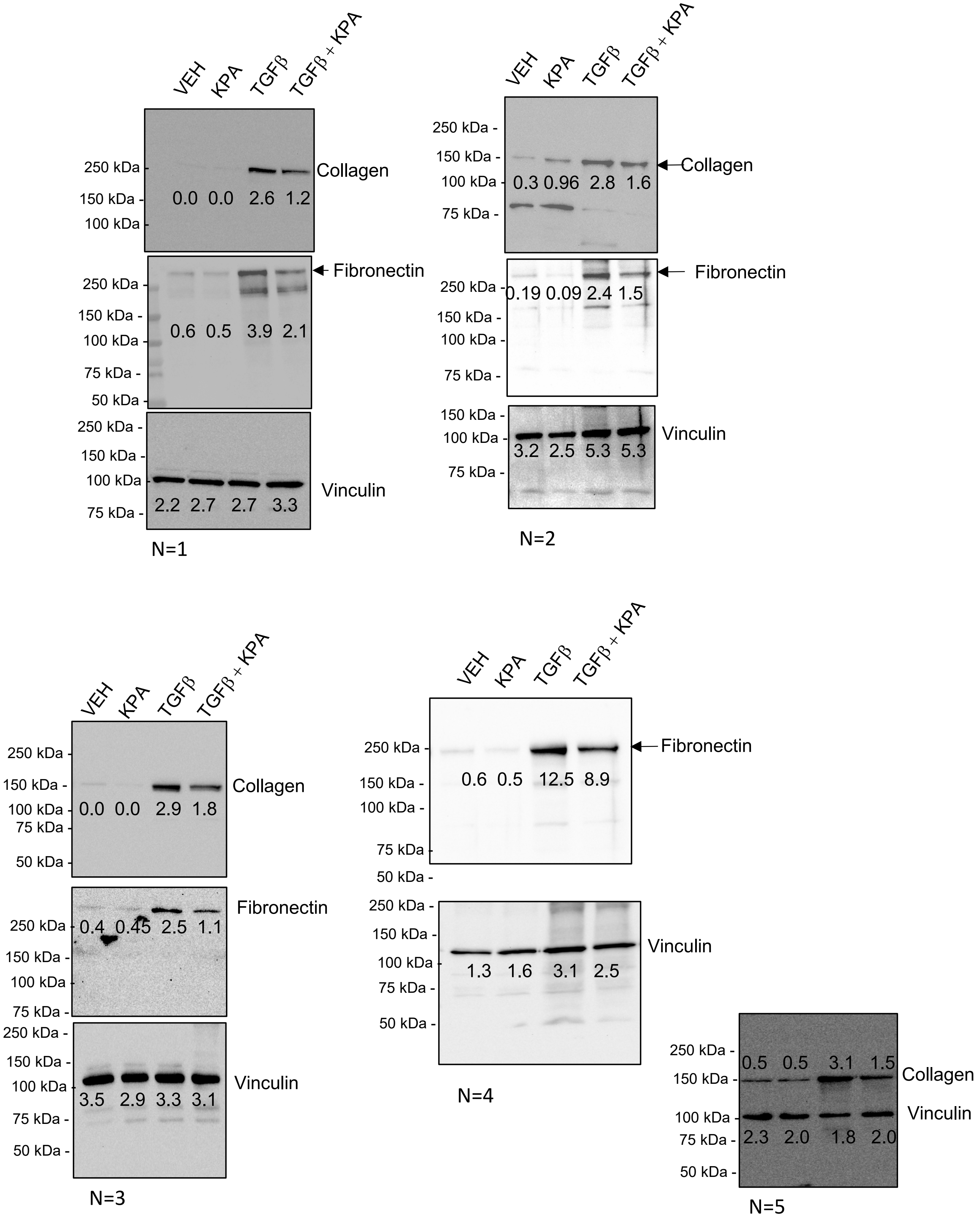

Prasad et al., BLOTS FIGURE 10A

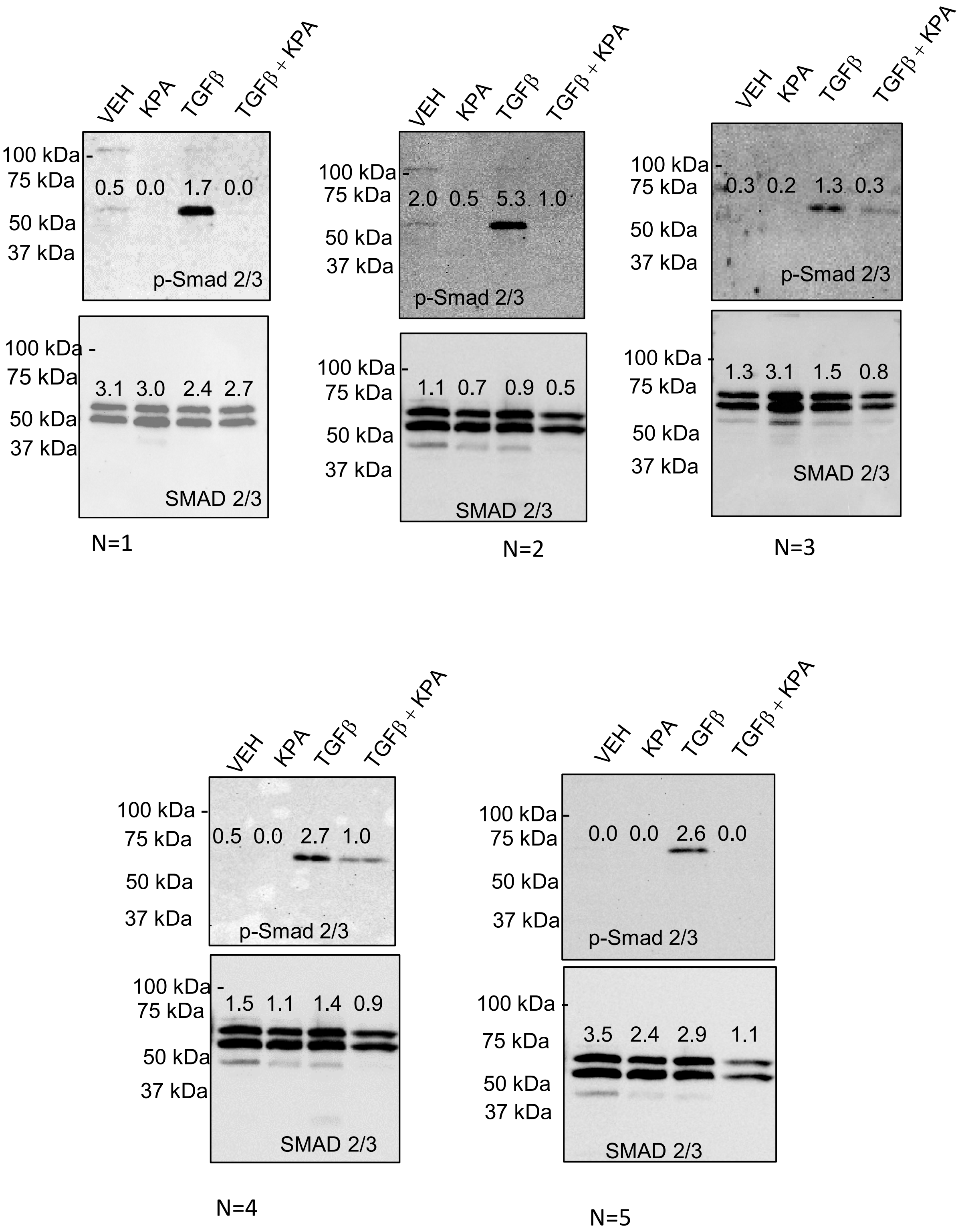

Prasad et al., BLOTS FIGURE 10B

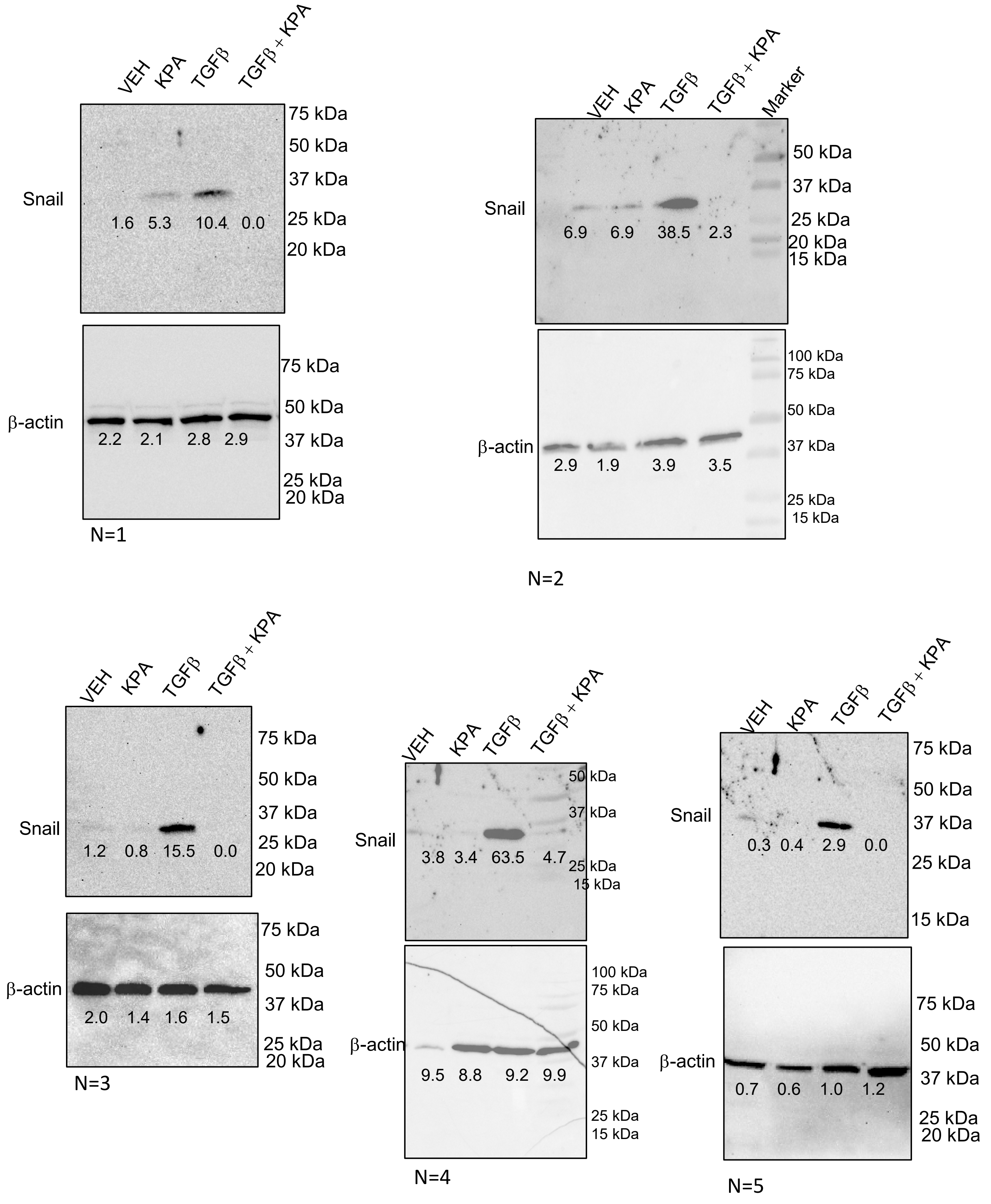

Prasad et al., Full unedited gels FIGURE 10D

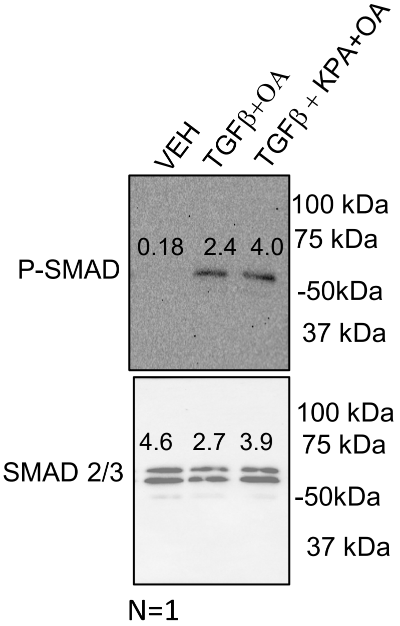

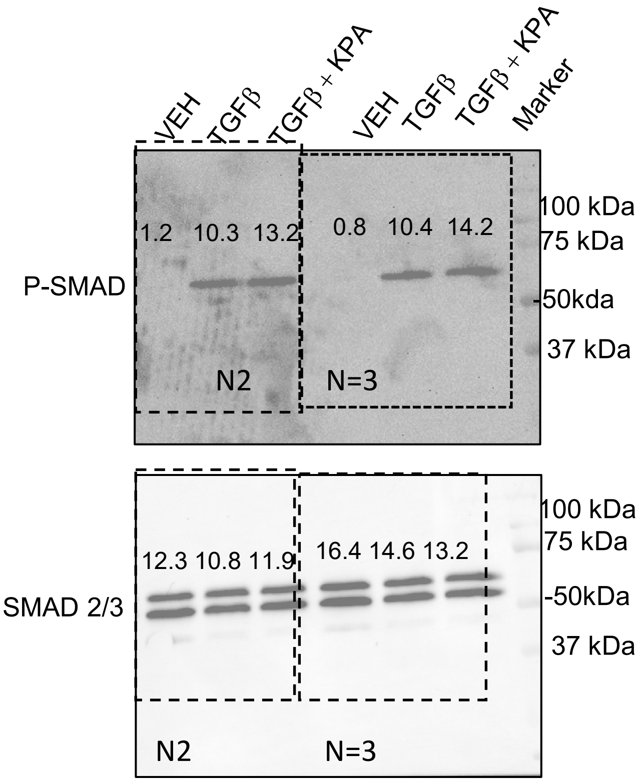

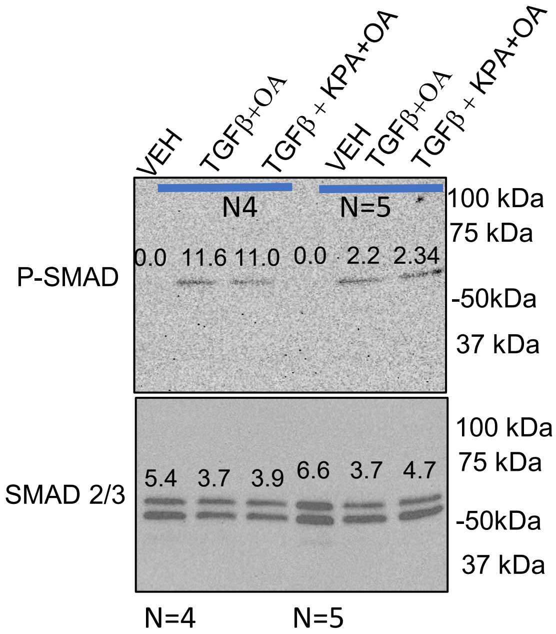

Supplement: Supplementary file 1 [file cells-13-01651-s001.zip › PRASAD full gels-9-30-24.pdf]
