# Supplementary figures and images for: Kisspeptin Alleviates Human Hepatic Fibrogenesis by Inhibiting TGFβ Signaling in Hepatic Stellate Cells
Source: Cells. 2024 Oct 4;13(19):1651. doi: 10.3390/cells13191651 (PMC11476267; doi:10.3390/cells13191651)

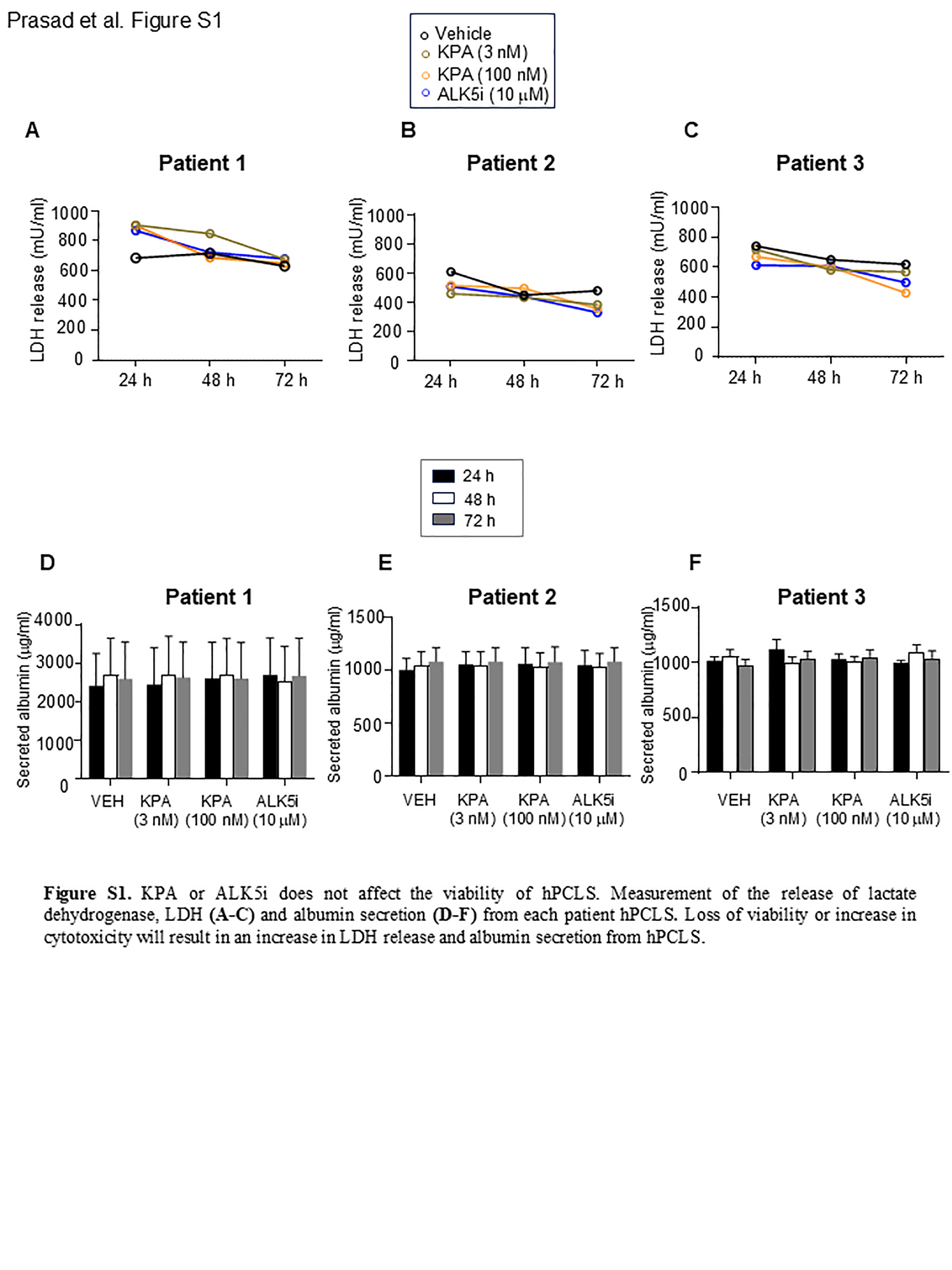

Supplement: Supplementary file 1 [file cells-13-01651-s001.zip › FIG S1.jpg]

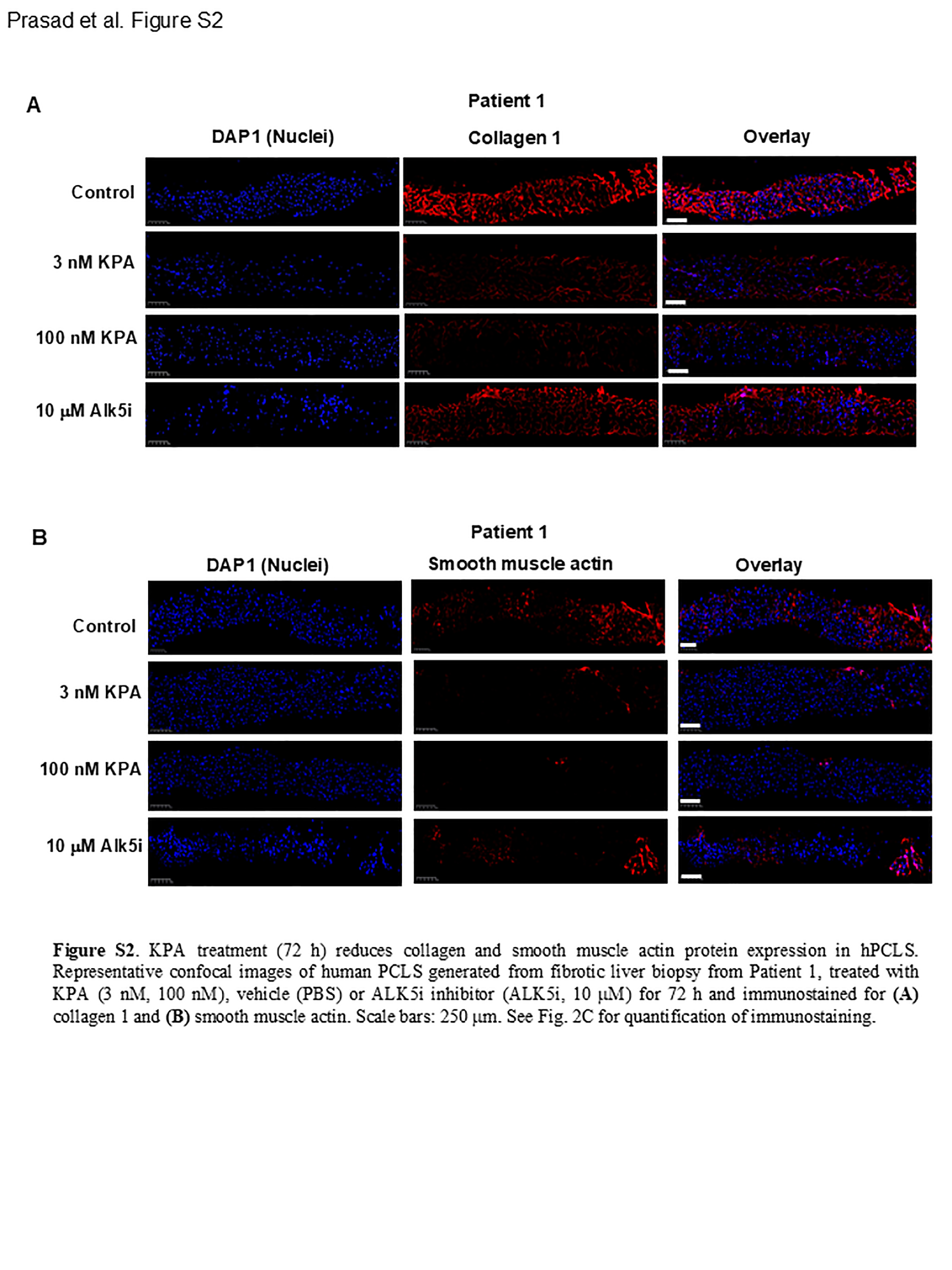

Supplement: Supplementary file 1 [file cells-13-01651-s001.zip › FIG S2.jpg]

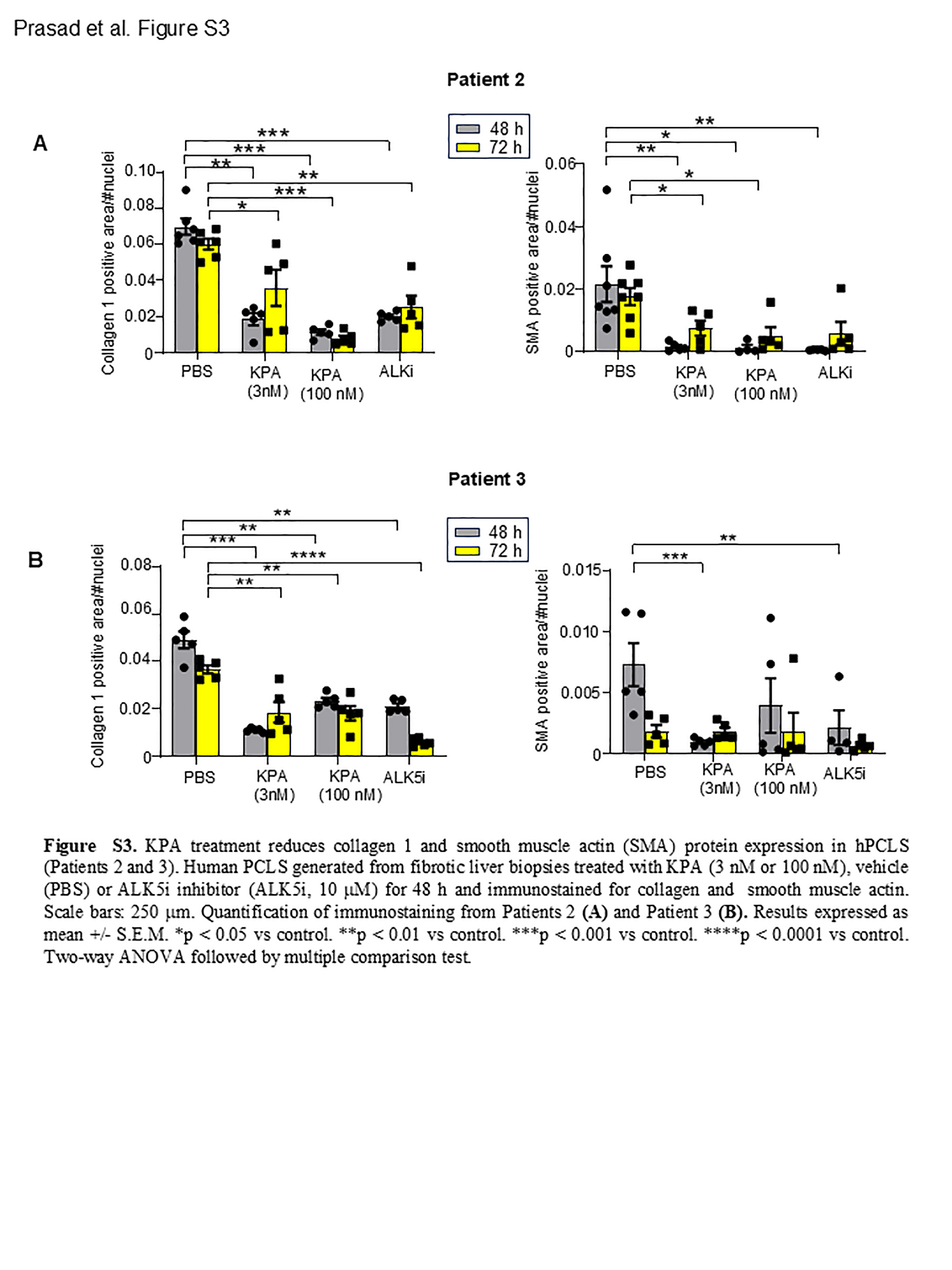

Supplement: Supplementary file 1 [file cells-13-01651-s001.zip › FIG S3.jpg]

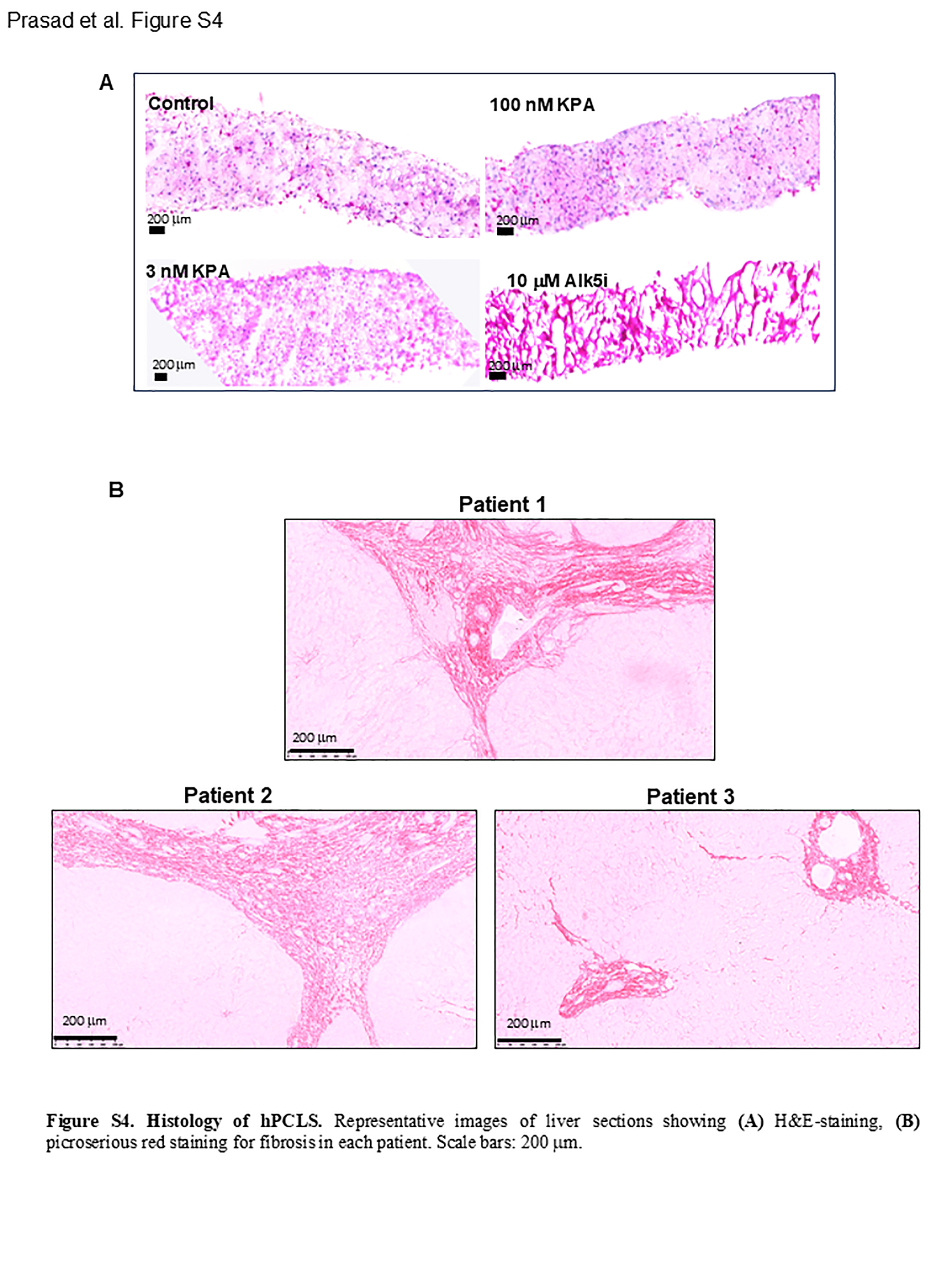

Supplement: Supplementary file 1 [file cells-13-01651-s001.zip › FIG S4.jpg]

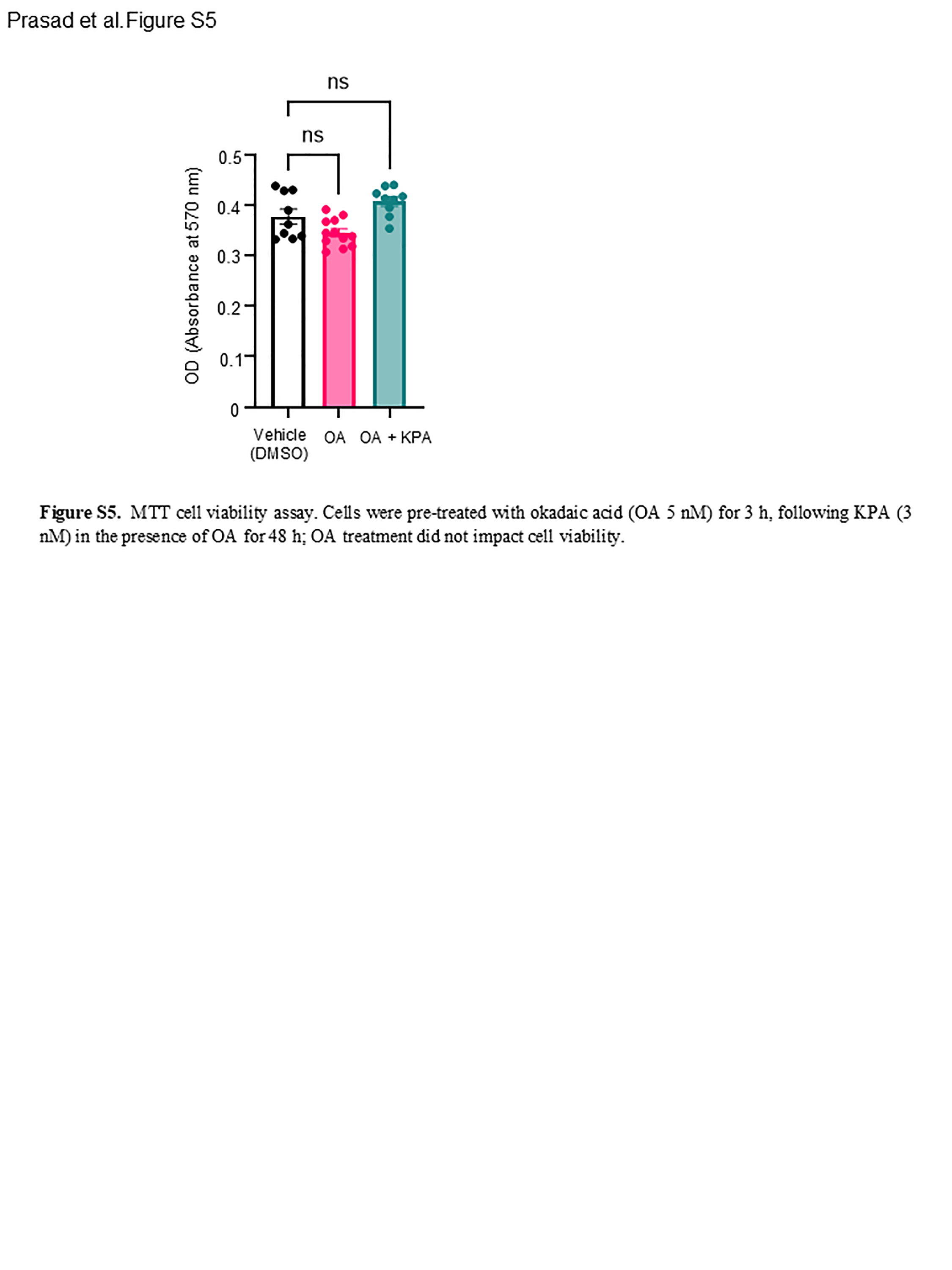

Supplement: Supplementary file 1 [file cells-13-01651-s001.zip › FIG S5.jpg]
